# Methodological considerations for identifying multiple plasma proteins associated with all-cause mortality in a population-based prospective cohort

**DOI:** 10.1038/s41598-021-85991-z

**Published:** 2021-03-24

**Authors:** Isabel Drake, George Hindy, Peter Almgren, Gunnar Engström, Jan Nilsson, Olle Melander, Marju Orho-Melander

**Affiliations:** 1 Diabetes and Cardiovascular Disease—Genetic Epidemiology, Department of Clinical Sciences in Malmö, Lund University, Clinical Research Centre House 60 Floor 13, Jan Waldenströms gata 35, 205 02 Malmö, Sweden; 2 Department of Population Medicine, College of Medicine Qatar University, Doha, Qatar; 3 Hypertension and Cardiovascular Disease, Department of Clinical Sciences in Malmö, Lund University, Malmö, Sweden; 4 Cardiovascular Epidemiology, Department of Clinical Sciences in Malmö, Lund University, Malmö, Sweden; 5 Experimental Cardiovascular Research, Department of Clinical Sciences in Malmö, Lund University, Malmö, Sweden

**Keywords:** Proteomic analysis, Machine learning, Predictive markers, Cancer, Cardiovascular diseases, Computational models, Biomarkers, Epidemiology, Outcomes research

## Abstract

Novel methods to characterize the plasma proteome has made it possible to examine a wide range of proteins in large longitudinal cohort studies, but the complexity of the human proteome makes it difficult to identify robust protein-disease associations. Nevertheless, identification of individuals at high risk of early mortality is a central issue in clinical decision making and novel biomarkers may be useful to improve risk stratification. With adjustment for established risk factors, we examined the associations between 138 plasma proteins measured using two proximity extension assays and long-term risk of all-cause mortality in 3,918 participants of the population-based Malmö Diet and Cancer Study. To examine the reproducibility of protein-mortality associations we used a two-step random-split approach to simulate a discovery and replication cohort and conducted analyses using four different methods: Cox regression, stepwise Cox regression, Lasso-Cox regression, and random survival forest (RSF). In the total study population, we identified eight proteins that associated with all-cause mortality after adjustment for established risk factors and with Bonferroni correction for multiple testing. In the two-step analyses, the number of proteins selected for model inclusion in both random samples ranged from 6 to 21 depending on the method used. However, only three proteins were consistently included in both samples across all four methods (growth/differentiation factor-15 (GDF-15), N-terminal pro-B-type natriuretic peptide, and epididymal secretory protein E4). Using the total study population, the C-statistic for a model including established risk factors was 0.7222 and increased to 0.7284 with inclusion of the most predictive protein (GDF-15; P < 0.0001). All multiple protein models showed additional improvement in the C-statistic compared to the single protein model (all P < 0.0001). We identified several plasma proteins associated with increased risk of all-cause mortality independently of established risk factors. Further investigation into the putatively causal role of these proteins for longevity is needed. In addition, the examined methods for identifying multiple proteins showed tendencies for overfitting by including several putatively false positive findings. Thus, the reproducibility of findings using such approaches may be limited.

## Introduction

Circulating biomarkers have the potential to improve risk stratification and targeted prevention strategies. For complex diseases, multiple biological processes and functional pathways regulate protein expression. This might explain why the expected clinical utility of novel biomarkers for disease outcomes often remain limited^1,2^. Advances in methodology empower exploratory analyses that aim to identify multiple protein biomarkers associated with a range of disease outcomes^3,4^. However, multiple testing, weak associations, and multicollinearity poses particular statistical challenges. Prediction models derived using time-to-event data typically rely on the Cox proportional hazards model^5^. To prevent overfitting and remove redundant variables, analysts may select predictors using stepwise selection (e.g. backward or forward elimination). Various extensions to the Cox model have however been developed to handle the particular setting of multicollinearity in high-dimensional models, where ridge, elastic net, and Least Absolute Shrinkage and Selection Operator (Lasso) are among the more often used^6,7^. In recent years, various machine learning algorithms have also been proposed as alternatives for modelling survival data^8–12^. Machine learning find the best-fitting model through automated processes that detect patterns that may include non-linear associations as well as interactions between variables, without the need for pre-specification by the researchers. Random survival forests (RSF) is a direct extension of the random forest method^12^ and has been implemented in clinical epidemiological settings^13–18^. Adequate identification and risk stratification of individuals with reduced life expectancy, especially in the middle-aged to elderly population, is an important public health priority and a central issue in clinical decision making. All-cause mortality is commonly used as a definite (hard) endpoint in studies of clinical risk factors also for specific disease events such as e.g. coronary artery disease. The objectives of this study were therefore to examine the associations between 138 plasma proteins and all-cause mortality, and to examine the potential usefulness of measuring multiple proteins by assessing the prediction improvement by adding a single versus multiple proteins to models for overall survival in a general population-based setting. In addition, we wanted to examine the reproducibility of different methods commonly used for identifying multiple predictors. Using a two-step random-split design, we examined the likelihood of chance findings by comparing four methods including Cox regression, stepwise Cox regression with backward elimination, Lasso-Cox regression, and RSF with backward elimination.

## Methods and subjects

### Study population

The Malmö Diet and Cancer Study (MDCS) is a population-based prospective cohort study established between 1991 and 1996^19^. Detailed descriptions of the cohort and representability has been published previously^20–22^. All men and women born between 1923–1950 and living in Malmö (Sweden) were invited to join. With a participation rate of approximately 40%, the cohort consists of 30,446 participants aged 44–73 years at baseline. Between October 1991 and February 1994, every other MDCS participant was invited to join a sub-study on cardiovascular disease risk (MDCS-cardiovascular arm (CVA); N = 6103)^23^. Participants in the MDCS-CVA donated fasting blood samples at baseline^24^. After protein quality control and exclusion of individuals based on pre-specified criteria, the final study population included 3918 subjects (Supplement Fig. 1**)**.

The study complies with the Declaration of Helsinki. All participants provided written informed consent, and the study was approved by the Ethics Review Committee at Lund University (LU 51-90).

### Proteomic profiling

Plasma proteins were analyzed using the Proseek Multiplex Oncology I, Version 2.1 and the Proseek Multiplex CVD I (Olink Bioscience) at the Science for Life Laboratory (SciLifeLab) in Uppsala, Sweden. Fasting blood samples taken at the baseline examination were separated into plasma and stored at − 80 °C. Plasma samples of 1 µL per participant were analyzed by the SciLifeLab using the Proseek assays. The proximity extension assay technique has been described in detail previously^3,25^. In short, the Proseek assay uses oligonucleotide-labeled antibody probe pairs that bind to their respective protein antigens in the plasma sample and uses DNA polymerase to form a PCR template. The individual DNA sequences were detected and quantified using specific primers by microfluidic real-time quantitative PCR chip (96.96, Dynamic Array IFC, Fluidigm Biomark). The chip was run with a Biomark HD instrument. A pre-processing normalization procedure for raw Proseek data was performed using Olink Wizard for GenEx (Multid Analyses, Sweden). For each data point, normalization for technical variation was performed by subtracting of the quantification cycle (C_q_) value in that well for the extension control^25^. An inter-plate control (IPC) was used to control for variation between plates. Normalization between runs was performed by subtracting the median IPC C_q_ from all the extension control-adjusted values on a plate, resulting in normalized protein expression (NPX, log_2_ scale) values accounting for potential batch effects. The NPX were finally adjusted to give a background noise level (based on negative controls) of around zero^25^. General calibrator curves as well as detailed technical information about the assays are available on the Olink homepage (http://www.olink.com). Limit of detection (LOD) was defined as 3 × standard deviations (SD) above the background noise based on the negative controls in each run. Eleven proteins were excluded due to > 50% missing values as determined by values below LOD. To retain a sufficient sample size with data on the remaining proteins, protein levels below the protein-specific LOD were imputed with LOD/2 among subjects with missing values. After quality control and imputation, a total of 138 plasma proteins remained for analysis. In order to provide comparable effect estimates across identified proteins, all protein levels (measured in arbitrary units) were subsequently ln-normalized and adjusted for age in a linear regression model and standardized to a mean of zero and a standard deviation of one. To further assess potential unaccounted for batch effects, we conducted principal component analyses (Supplement Fig. 2–6) (i.e. to assess individual differences by plate) in the total study population, two random-split samples of the study population, in a smaller random sample of the total study population (N = 200) as well as in all subjects with no imputed protein levels (N = 772); no visually significant batch effects were observed. A flow chart of participants including quality control steps and imputation is found in Supplement Fig. 1 and a description of the included proteins including mean (range) NPX and the number of subjects with imputed values is shown in Supplement Table 1.

### Clinical outcomes

The study population was followed from their baseline examination until 31 December 2014, death, or emigration. Vital status and underlying causes of death were obtained by linkage to the Swedish Tax Agency and the Swedish Cause of Death Registry. When a death occurs, this event is registered at the civil registry system with information on the date and cause of death based on the codes used in the International Classification of Disease (ICD) Version 9 and 10. As our study investigates all-cause mortality, all deaths were included as the endpoint independent of the underlying cause of death.

### Clinical risk factors

Information on age and gender were extracted from the subjects’ Swedish personal identification number. Smoking status was classified as never, former, or current smokers. Educational level was categorized as elementary, junior high school, high school, continued education, or university/college degree. Direct measurements taken by trained nurses included height (cm) and weight (kg), which was used to calculate body mass index (BMI; kg/m^2^). Blood pressure was measured after 5 min of supine rest. History of hypertension was defined as a blood pressure at baseline above 140/90 mmHg and/or reported use of anti-hypertensive treatment in the baseline questionnaire. Prevalent diabetes mellitus (yes/no) was confirmed by diagnosis in local or national registries or having a fasting whole blood glucose value of > 6.0 mmol/L at the baseline screening. During screening, HbA1c (%), triglycerides (mmol/L), and high-density lipoprotein cholesterol (HDLC; mmol/L) were measured at the Department of Clinical Chemistry, Skåne University Hospital in Malmö. Low-density lipoprotein cholesterol (LDLC) was estimated using Friedewald’s formula. Blood samples stored at − 80 °C were used for analyses of high-sensitivity C-reactive protein (hsCRP) using the Tina-quant CRP latex high sensitivity assay (Roche Diagnostics, Basel, Switzerland) on an ADIVA 1650 Chemistry System (Bayer Healthcare, NY, USA).

### Statistical analysis

The Pearson’s correlation coefficients for all proteins were visualized with a heat map matrix ordered with hierarchical clustering (Supplement Fig. 7). To identify single proteins associated with all-cause mortality, a Cox proportional hazards regression with follow-up time as the time-scale was used. Clinical covariates included age, sex, smoking status, BMI, educational level, history of hypertension, prevalent diabetes mellitus, C-reactive protein, HbA1c, and LDL-cholesterol. HbA1c and hsCRP were ln-transformed to normalize distribution. The Bonferroni method was used to account for multiple testing (P-value = 0.05/138 proteins). To discriminate for the usefulness of the individual proteins for mortality prediction we calculated Harrell’s C-statistic (concordance index), category-free net reclassification improvement (cNRI) and integrated discrimination improvement (IDI)^26^.

To identify multiple proteins that robustly associate with all-cause mortality after mutual adjustment, the study population was randomly split into two samples to simulate a discovery and replication cohort. Randomization was performed by sorting on underlying cause of death as a grouping variable to assure equal distribution of causes of death in the two samples. Differences in baseline characteristics were examined using ANOVA and Chi-square test. We performed the two-step random-split analysis using four methodological approaches. Firstly, we repeated single protein analyses with adjustment for covariates and retained proteins that were nominally associated with all-cause mortality in both random samples. Secondly, we ran a stepwise Cox regression with backward elimination of proteins with P-values > 0.05 and with forced inclusion of covariates. Thirdly, we used a Lasso-Cox regression with tenfold cross-validation, maximization of Cox model partial likelihood and model selection based on lambda-minimum. Covariates were forced into the model. For all Cox regression models, we assumed linear associations between proteins and mortality and no protein–protein interactions. The scaled Schoenfeld residuals were used to test the proportional hazards assumption; no deviations were noted. Finally, we applied a RSF backward algorithm. The method has been described in detail previously^16^. For evaluation of the RSF procedure, three different models were computed including: (1) covariates only, (2) covariates and all proteins, and (3) covariates and proteins selected using the backward elimination procedure. For each RSF model, 100 repetitions were computed and used to calculate means and 95% CIs of prediction error rates. The prediction error rate corresponds to 1 minus the C-index, where a lower value corresponds to better prediction^16^. The default values for computation of RSFs were used. Each RSF was computed using 1000 bootstrap samples and the log-rank splitting rule with 10 splits per variable.

Finally, we included proteins selected concordantly in both random samples in Cox regression models using the full study population. To quantify the predictive performance of the multiple protein models, we used Harrell’s C-statistic and compared these models with a clinical model (i.e. established risk factors only) as well as the clinical model with inclusion of only the strongest protein biomarker using the likelihood ratio (LR) test. For single proteins significantly associated with mortality (i.e. after Bonferroni correction) as well as for proteins concordantly selected across both random samples and using all examined methodological approaches, we constructed protein scores using the quintile ranking of participants based on their plasma protein levels and estimated 10-year absolute risk of mortality across quintiles of scores. Analyses were conducted with the R Version 3.5.1 (The R Project for Statistical Computing, Vienna, Austria), including the *randomForestSRC* package for RSF analysis and the *glmnet* package for Lasso-Cox regression, and Stata/SE Version 14.2 (StataCorp, College Station, TX, USA).

## Results

### Description of study population

**Figure 1:**
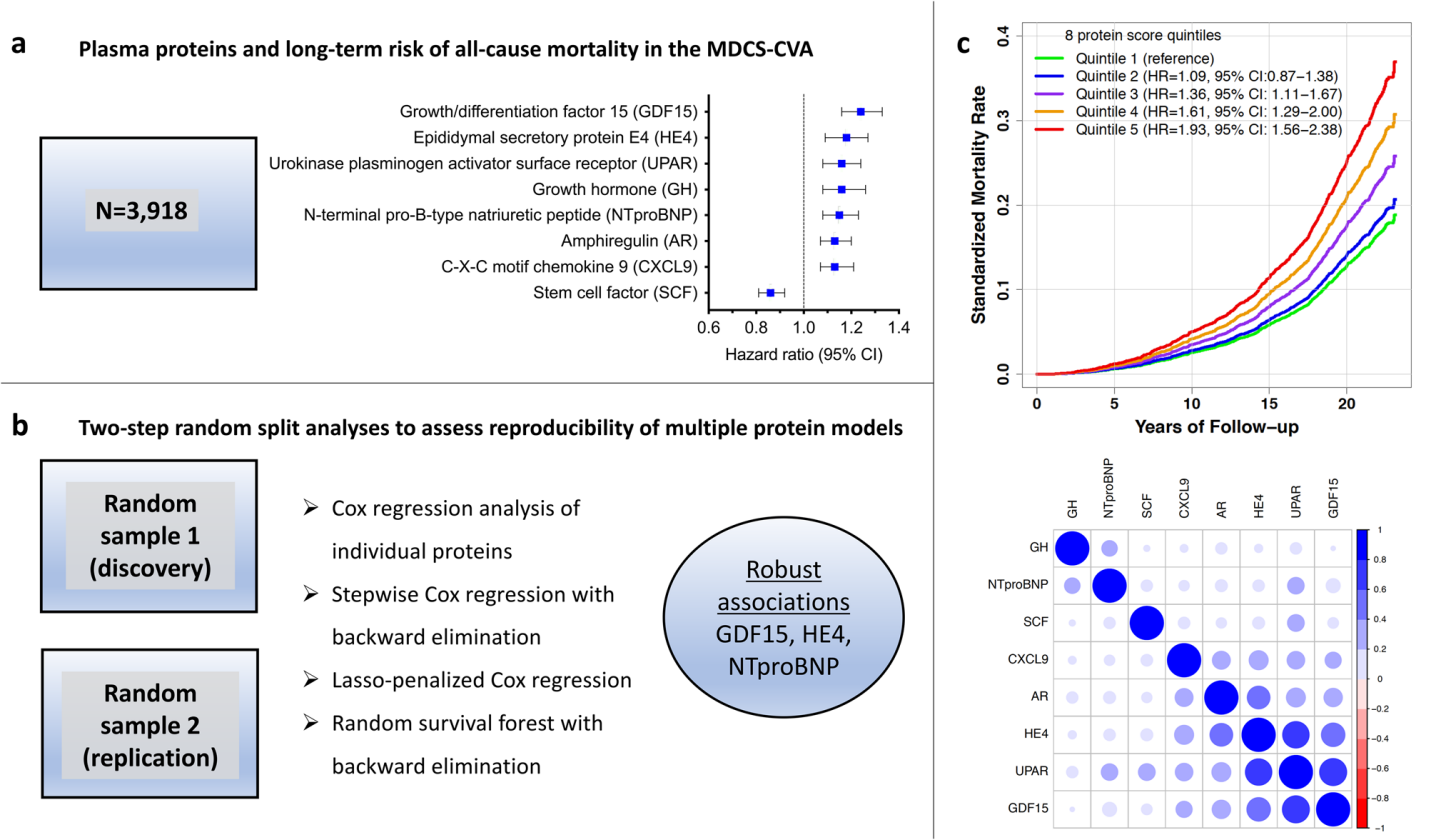
Summary of study design, methods used, and main results. (**a**) Individual proteins found to associate with all-cause mortality in the Malmö Diet and Cancer Study Cardiovascular Arm (MDCS-CVA) using Cox regression analysis adjusting for established risk factors (**b**) Two-step random split analyses to assess the reproducibility of multiple protein models defined using four methodological approaches. Three proteins were robustly replicated across the random samples and all included methods. (**c**) The association between a plasma protein score including the proteins in section (a) with all-cause mortality and the between-protein Pearson correlation coefficients.

A summary of the study design and main results is shown in Fig. 1. Baseline characteristics of the study population are shown in Table 1. Overall, there were no differences in the examined characteristics between the two random samples of the study population, except for higher hsCRP levels in random sample 1 compared to random sample 2 (P = 0.04). During a median follow-up of 21.7 years (interquartile range 20.9–22.4 years), there were 974 deaths from any cause. There was a positive correlation structure between several of the investigated proteins (Supplement Fig. 7).

**Table 1:** Description of the MDCS-CVA and two random samples of the study population.

Characteristic	Total study population	Random sample 1	Random sample 2	P-value*
Number of subjects	3918	1963	1955	
Number of deaths	974	491	483	
**Cause of death (%)**				
Cancer	41.8	41.6	42.0	
Cardiovascular	29.1	28.9	29.2	
Neurological	6.1	6.1	6.0	
Respiratory	5.8	5.7	5.8	
Age, years	57.2 (5.9)	57.4 (5.9)	57.1 (5.9)	0.17
Male gender (%)	40.4	41.9	38.8	0.05
Body mass index, kg/m^2^	25.6 (3.9)	25.6 (3.8)	25.5 (4.0)	0.62
Current smoking (%)	25.8	27.2	24.5	0.12
University/college degree (%)	11.8	11.6	12.1	0.95
Prevalent diabetes mellitus (%)	3.5	3.4	3.5	0.84
History of hypertension (%)	61.9	61.5	62.3	0.60
C-reactive protein, mg/dL^#^	1.3 (0.7–2.7)	1.4 (0.7–2.8)	1.3 (0.6–2.7)	0.04
LDL-cholesterol, mmol/L	4.2 (1.0)	4.2 (1.0)	4.2 (1.0)	0.09
HbA1c, %^#^	4.8 (4.5–5.1)	4.8 (4.5–5.1)	4.8 (4.5–5.1)	0.94

*Mean (standard deviation, SD) are shown unless otherwise noted. Chi-square test (categorical variables) and ANOVA (continuous variables) used to test differences in characteristics between random sample 1 and 2.

^#^Median (interquartile range) and P-value from ANOVA using ln-transformed variable due to non-normal distribution.

### Individual proteins associated with all-cause mortality

**Figure 2:**
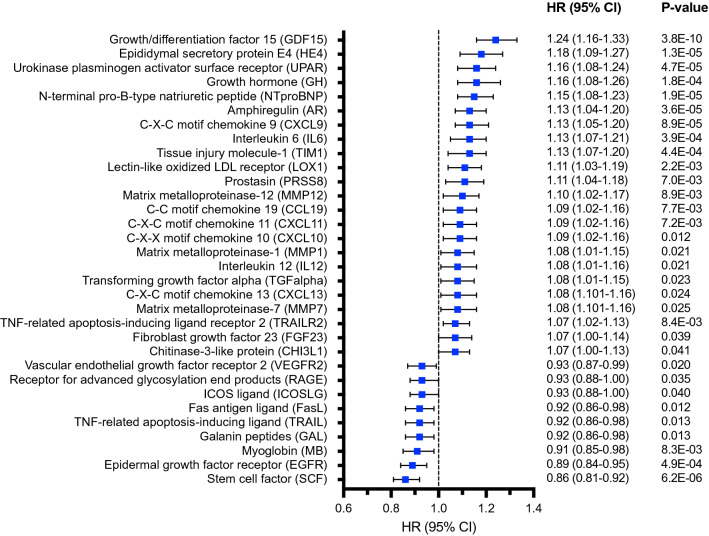
Forest plot of plasma proteins associated with risk of all-cause mortality with nominal significance level (P < 0.05) after covariate adjustment in the MDCS-CVA (n = 3,918). Hazard ratio (HR) and 95% confidence interval (CI) per standard deviation (SD) from a Cox regression model adjusted for age, sex, smoking status, BMI, educational level, history of hypertension, prevalent diabetes mellitus, C-reactive protein (ln-transformed), HbA1c (ln-transformed), and LDL-cholesterol. Bonferroni corrected significance threshold was P < 0.00036.

The analyses of the individual proteins in relation to all-cause mortality in the total study population is shown in Supplement Table 2. With adjustment for covariates, 32 proteins were nominally associated with all-cause mortality (P < 0.05) (Fig. 2). After Bonferroni correction, eight proteins remained significantly associated with all-cause mortality (Fig. 2; Table 2). These included amphiregulin (AR), C-X-C motif chemokine 9 (CXCL9), epididymal secretory protein E4 (HE4), growth hormone (GH), growth/differentiation factor-15 (GDF15), N-terminal pro-B-type natriuretic peptide (NTproBNP), stem cell factor (SCF), and urokinase plasminogen activator receptor (UPAR). Compared to a clinical model (i.e. with established risk factors only) all identified proteins improved discrimination of all-cause mortality as assessed by the C-statistic and comparing model discrimination using the LR test (all P < 0.001). In addition, there was a significant improvement in IDI and cNRI for all markers, except for NTproBNP, where no improvement in cNRI was observed (P = 0.12).

**Table 2:** Performance metrics of individual plasma proteins in addition to clinical covariates for prediction of all-cause mortality in the MDCS-CVA.

Model	C-statistic	P-value (LR)	IDI	P-value	cNRI	P-value
Clinical model*	0.7222	–			–	
+ AR	0.7247	0.0001	0.0039	0.0001	0.1673	< 0.0001
+ CXCL9	0.7243	0.0002	0.0033	0.0008	0.1439	< 0.0001
+ HE4	0.7260	< 0.0001	0.0058	< 0.0001	0.1788	< 0.0001
+ GH	0.7248	0.0001	0.0042	0.0002	0.1268	0.0006
+ GDF15	0.7284	< 0.0001	0.0088	< 0.0001	0.1287	0.0005
+ NTproBNP	0.7235	< 0.0001	0.0044	0.0003	0.0569	0.1238
+ SCF	0.7253	< 0.0001	0.0057	0.0001	0.1419	0.0001
+ UPAR	0.7266	< 0.0001	0.0040	0.0002	0.1564	< 0.0001

*LR* likelihood ratio test, *cNRI* category-free Net Reclassification Improvement, *IDI* integrated discrimination improvement.

*Cox regression model including age, sex, smoking status, BMI, educational level, history of hypertension, prevalent diabetes mellitus, C-reactive protein (ln-transformed), HbA1c (ln-transformed), and LDL-cholesterol.

### Selection of multiple predictors using regression-based methods and RSF

Nine proteins were associated with all-cause mortality in both random samples of the study population (P < 0.05), including AR, CXCL9, GDF15, GH, HE4, NTproBNP, SCF, and UPAR, and additionally interleukin-6 (IL6), which did not reach the threshold for significance in the full study population after Bonferroni correction for multiple testing (Table 3). Results for the single protein analyses in the two random samples are shown in Supplement Table 3. Using a stepwise Cox regression with backward elimination resulted in 21 proteins retained in random sample 1 and 16 proteins retained in random sample 2. Out of these, 7 proteins were retained in both random samples, including GDF15, HE4, NTproBNP, caspase-3 (CASP3), epidermal growth factor receptor (EGFR), ezrin (EZR), and myeloperoxidase (MPO). However, MPO showed diverging associations in the two random samples (data not shown). In a Lasso-Cox regression, 26 proteins were retained in random sample 1 and 27 proteins in random sample 2. Out of these, 13 proteins were retained in both random samples, including CXCL9, EGFR, EZR, GDF15, GH, HE4, kallikrein-6 (KLK6), myoglobin (MB), NTproBNP, SCF, tissue-injury molecule-1 (TIM), and TNF-related apoptosis-inducing ligand (TRAIL) (data not shown). The RSF backward algorithm identified 49 and 30 proteins in random sample 1 and 2, respectively. Out of these, 21 proteins were retained in both random samples (data not shown). Compared to a RSF model with only covariates and covariates together with all proteins, the RSF models with covariates and selected proteins had lower mean prediction error rates in both random samples, as well as in the full study population including the 21 proteins retained in both random samples (Supplement Table 4).

**Table 3:** Performance metrics of different Cox regression models where proteins were selected for inclusion by using a two-step random-split approach in the MDCS-CVA (N = 3,918).

Model	C-statistic	Change in C-statistic from clinical model	P-value (LR)	Change in C-statistic from clinical + strongest marker model	P-value (LR)
Clinical model*	0.7222	–	–		
Clinical + strongest marker**	0.7284	0.0062	< 0.0001	–	–
Clinical + all proteins***	0.7705	0.0483	< 0.0001	0.0421	< 0.0001
Clinical + Cox^a^	0.7379	0.0157	< 0.0001	0.0095	< 0.0001
Clinical + StepwiseCox^b^	0.7373	0.0151	< 0.0001	0.0089	< 0.0001
Clinical + LassoCox^c^	0.7492	0.0270	< 0.0001	0.0208	< 0.0001
Clinical + RSF^d^	0.7436	0.0241	< 0.0001	0.0152	< 0.0001

*Covariates included in the model were age, sex, smoking status, BMI, educational level, history of hypertension, prevalent diabetes mellitus, C-reactive protein, HbA1c, and LDL-cholesterol.

**Covariates and growth/differentiation factor-15 (GDF-15).

***Covariates and all proteins (n = 138).

^a^Covariates and 9 proteins (AR, CXCL9, GDF15, GH, HE4, IL6, NTproBNP, SCF, UPAR) associated (P < 0.05) with all-cause mortality in a Cox regression model after adjustment for covariates in both random samples of the MDCS-CVA.

^b^Covariates and 6 proteins (GDF15, CASP3, EGFR, EZR, HE4, NTproBNP) associated (P < 0.05) with all-cause mortality with mutual adjustment in both random samples of the MDCS-CVA using a stepwise Cox regression with backwards elimination of proteins with P < 0.05. MPO was excluded due to diverging associations with all-cause mortality in the two random samples.

^c^Clinical variables and 13 proteins (CXCL9, EGFR, EZR, GDF15, GH, HE4, KLK6, MB, NTproBNP, SCF, TIM, TRAIL, UPAR) retained in both random samples of the MDCS-CVA using a Lasso penalized Cox regression and lambda minimum for protein selection.

^d^ Clinical variables and 21 proteins (FABP4, FasL, GDF15, HE4, HGF, IL12, IL6, mAmP, MMP1, MMP12, MYD88, NTproBNP, PRSS8, PSGL1, PTPN22, PTX3, RAGE, REN, SCF, THPO, TIM) retained in both random samples using a RSF backward elimination approach.

### Performance metrics of multiple protein models

A summary of findings using the two-step random-split approach to identify multiple protein models is shown in Supplement Table 5. In total, only three proteins were consistently retained across all four methods and in both random samples. These included HE4, GDF15 and NTproBNP. We examined model discrimination by improvement in the C-statistic in models including multiple proteins selected using the two-stage random split analysis to a clinical model with established risk factors for mortality (Table 3). Compared to the clinical model, all models including one or more protein biomarkers showed an increase in the C-statistic (all P < 0.0001). The C-statistic for the clinical model was 0.7222, which increased to 0.7284 when including GDF15 (strongest marker) and to 0.7705 when including all proteins. The two Cox regression models where proteins were included on the basis of the individual protein analysis or the stepwise selection procedure performed similarly, with a C-statistic of 0.7379 and 0.7373, respectively. The models based on results from the Lasso-Cox and the RSF performed similar and better than the traditional Cox regression models with C-statistics of 0.7492 and 0.7436, respectively (Table 3).

We separated participants into quintiles on the basis of (1) GDF15 levels, (2) a score including three proteins consistently identified across different methodological approaches (GDF15, HE4, and NTproBNP) and (3) a score including eight proteins that were associated with all-cause mortality after Bonferroni correction (GDF15, HE4, NTproBNP, AR, CXCL9, GH, UPAR, and SCF). Supplement Fig. 9 shows the cumulative hazard rate for all-cause mortality across quintiles of the biomarker scores. The HRs in quintile five compared to quintile 1 were 1.48 (95% CI 1.20–1.84) for GDF15, 1.67 (95% CI 1.33–2.08) for the 3-protein score, and 1.93 (95% CI 1.56–2.38) for the eight-protein score. For the 8-protein score, the HR corresponded to a 10-year absolute mortality risk of 4.9 (95% CI 4.0–5.8) in quintile 5 compared to 2.5 (95% CI 2.0–3.0) in quintile 1 (Supplement Fig. 10).

## Discussion

Based on explorative analyses of 138 plasma proteins, we identified eight proteins associated with all-cause mortality after adjustment for known clinical risk factors. Compared to the clinical model, all of the identified proteins significantly improved prediction of all-cause mortality, however, the increase in the C-statistic was modest. Comparing four methods for selecting multiple predictors, there were only three protein biomarkers that showed consistent model inclusion across all four approaches (HE4, GDF15, and NTproBNP). Notably, the multivariable methods examined in this study showed tendencies for overfitting and limited robustness in selecting multiple predictors based on the results from the two-step random split analysis.

All-cause mortality is a heterogeneous endpoint and as such most of the identified proteins are known to have pleiotropic functions with involvement in a range of conditions and diseases. However, while some of the proteins are positively correlated, there are no established links between the identified proteins, suggesting that they may act fairly independently on development of disease and future risk of mortality (Supplement Fig. 8). GDF-15 (also known as macrophage inhibitory cytokine-1) is a stress response cytokine and a member of the transforming growth factor-ß superfamily. In humans, increasing GDF-15 levels have been associated with inflammation, cardiovascular disease, type 2 diabetes, and cancer^25,27–29^. The established heart failure biomarker NTproBNP have been studied extensively in relation to risk of cardiovascular diseases^30^ and a meta-analysis found that NTproBNP levels also associate with all-cause mortality in the general population^31^. HE4 levels are elevated in patients with ovarian cancer^32^. A recent study also found that HE4 levels are elevated in patients with chronic heart failure and that levels predict heart failure outcome^33^. Compared to GDF-15 and NTproBNP, HE4 is much less studied in general populations using a longitudinal design. The soluble form of UPAR has been studied extensively with respect to disease outcomes, both in general populations and in patient populations^34^. UPAR is generally believed to be a marker of low-grade inflammation in the general population and is strongly affected by smoking^35^. CXCL9 is a cytokine with chemotactic functions that function as a ligand to the CXC chemokine receptor 3 expressed on T-lymphocytes and natural killer cells. CXCR3 and its ligands CXCL9/10/11 have been proposed to play an important role in recruitment of Th1 cells in atherosclerotic plaques^36^ and to have a more complex role in the tumor microenvironment^37^. A previous study within the MDCS-CVA showed that plasma SCF associates with decreased risk of both cardiovascular disease and all-cause mortality^38^. AR is a ligand to the epidermal growth factor receptor that has been shown to be pro-oncogenic, with functional studies implicating most of the cancer hallmarks^39^. AR has also been shown to be expressed by numerous immune cells in a variety of inflammatory conditions^40^. In a previous study within the MDCS-CVA, high-sensitivity GH was associated with increased risk of cardiovascular morbidity and mortality^41^. In cancer, GH, via its mediator peptide insulin-like growth factor-1 (IGF-1) is known to influence regulation of cellular growth^42^.

This is to the best of our knowledge the first large-scale investigation into multiple plasma proteins in relation to long-term risk of all-cause mortality in a population-based setting. There are several studies examining the usefulness of large-scale data such as various ‘omics’ data (including proteomics) for prediction of disease events in general populations or in patient populations^4,43–45^. Overall, few biomarkers have been robustly replicated in independent study populations as well as so far proven to be sufficiently useful for clinical implementation. For this reason, the findings from this study should be regarded as exploratory and in need of replication in independent study populations. In addition, the two-step random split analyses indicate that the potentially low information value on many of the included proteins as well as multi-collinearity may result in several false positive findings when using several commonly used multivariable approaches. Further, while several plasma proteins were found to robustly associate with all-cause mortality in this study population, the causal nature of these associations are not known. A recent large scale proteomics Mendelian randomization study reported some evidence for a causal positive association between GDF15 and body mass index/weight, and a causal inverse association between SCF and HDL cholesterol and a positive association with triglycerides^46^. As such it is likely that several of the investigated proteins represent pathways related to many of the established risk factors for mortality, which may explain the limited improvement in prediction of this outcome.

This study has several strengths and limitations. The main strengths include the prospective study design and the use of a well-characterized population-based cohort with information on key covariates and high completeness of endpoint ascertainment. Loss to follow-up due to emigration was less than 0.5%. In general, there was a rather large discrepancy between the identified proteins in the two random samples from the same study population, suggesting that probing large proteomics dataset is likely to include several false positive findings as well as producing over-fitted prediction models when using commonly implemented predictor selection methods. We aimed to overcome the heterogeneity of the study outcome by assuring equal distribution of the underlying causes of deaths in the two random samples, however, differences between the two random samples may be due to unaccounted for heterogeneity between samples. We could not identify any statistically significant differences in distribution by plate or imputed protein values between the two random samples. There appeared to be no major batch effects in the total study population or based on visual inspection of PCA plots in smaller random samples of the study population (see Supplement Fig. 2 and 5). However, based on visual inspection of PCA plots there appeared to be slight differences between the two random samples (see Supplement Fig. 3 and 4). We therefore repeated the main analyses with inclusion of plate number as a covariate, however, results were virtually unchanged (data not tabulated). While specific causes of death could be investigated as potential outcomes in this study, the statistical power for such analysis would be limited. The main rationale for our two-step random split approach was to provide a similar setting as to examining the same methodological approaches when using two independent cohorts (discovery and replication) i.e. a real-world type of scenario. Thus, the aim was not to perform a traditional examination of model robustness nor to validate the specific models (i.e. using a training and test set for model parameters). The key interest in our paper was rather to consider potential of chance findings (and thus reproducibility) when using these methods in two independent study populations. This type of approach is however not comparable to external replication because both samples were drawn from the same study population and thus analyses in the two samples share sources of bias. Overall, the two methods designed to maximize prediction (i.e. the Lasso-penalized Cox regression and the RSF method) retained a higher number of proteins compared to the traditional Cox regression methods where covariates selection is guided by statistical significance only. Accordingly, these models also performed better in predicting all-cause mortality. The RSF approach holds some advantages over the traditional regression-based approaches for exploratory analysis of complex datasets. For example, it is possible probe potential protein–protein interactions as well as examining non-linear associations to identify suitable protein level cut-points. For a specific outcome analysis of an exploratory nature, the RSF approach may thus be an appealing complement to the Cox regression approaches. Nevertheless, the results of such a model will, similarly to the other methods examined in this study, need replication in independent samples.

In conclusion, we identified several proteins that associated with all-cause mortality, however, the causal nature of these associations remains to be investigated. Exploratory multiple protein models may display poor replicability and should be interpreted as hypothesis-generating unless replicated in independent study populations.

## Supplementary Information

Supplementary Information

## Data Availability

The datasets analyzed during the current study are not publicly available due to restrictions in the ethical permission but the data can be accessed through the corresponding author upon reasonable request and with permission of the Malmö Diet and Cancer Study Steering Committee.
