# Supplementary material for: Methodological considerations for identifying multiple plasma proteins associated with all-cause mortality in a population-based prospective cohort
Source: Sci Rep. 2021 Mar 24;11:6734. doi: 10.1038/s41598-021-85991-z (PMC7990913; doi:10.1038/s41598-021-85991-z)
Supplement: Supplementary file 1 — Supplementary Information [file 41598_2021_85991_MOESM1_ESM.pdf]

Supplementary material

Methodological considerations for identifying multiple plasma proteins associated with all-cause mortality in a population-based prospective cohort

Authors: Isabel Drake, George Hindy, Peter Almgren, Gunnar Engström, Jan Nilsson, Olle Melander, Marju Orho-Melander

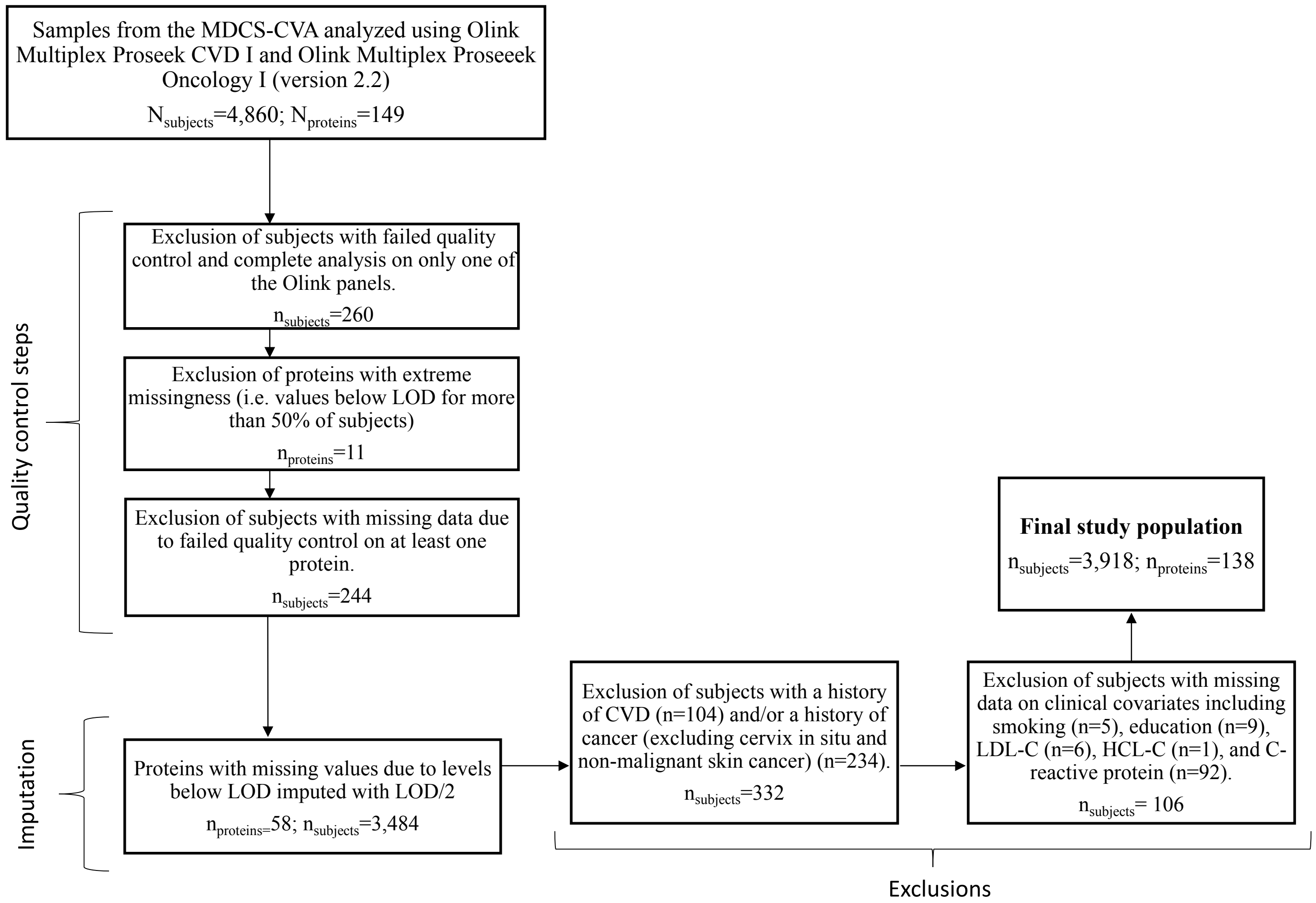

Supplement Figure 1. Flow chart of study participants including protein quality control steps, imputation, and study exclusions the Malmö Diet and Cancer Study Cardiovascular Arm (MDCS-CVA).

Supplement Table 1. Description of the 138 plasma proteins passing quality control in the MDCS-CVA (N=4356). Standardized mean values are in arbitrary units (AU). NA denotes that there are no subjects with imputed protein values.

Protein name (abbreviation)	Panel	Mean (range)	Limit of detection (LOD)	N imputed with LOD/2
Adrenomedullin (AM)	CVD/Onco	6.1 (2.9-8.1)	1.00	NA
Agouti-related protein (AGRP)	CVD	4.2 (2.3-7.4)	0.50	NA
Amphiregulin (AR)	Onco	1.8 (0.4-8.2)	0.76	29
Angiopoietin-1 receptor (TIE2)	CVD/Onco	6.1 (4.1-7.8)	1.40	NA
B-cell activating factor (BAFF)	Onco	6 (0.8-8.3)	1.53	1
C-C motif chemokine 19 (CCL19)	Onco	9.4 (6.7-13.4)	1.23	NA
C-C motif chemokine 20 (CCL20)	CVD	5.5 (2.1-11.8)	1.10	NA
C-C motif chemokine 3 (CCL3)	CVD	1.1 (0.1-7.7)	0.20	78
C-C motif chemokine 4 (CCL4)	CVD	7.2 (4.7-12.1)	1.20	NA
C-X-C motif chemokine 1 (CXCL1)	CVD	7.3 (4.2-10.4)	1.60	NA
C-X-C motif chemokine 10 (CXCL10)	Onco	7.0 (0.4-11.4)	0.40	1
C-X-C motif chemokine 11 (CXCL11)	Onco	4.1 (0.9-10.1)	1.72	6
C-X-C motif chemokine 13 (CXCL13)	Onco	8.1 (0.2-12.7)	0.49	88
C-X-C motif chemokine 5 (CXCL5)	Onco	9.9 (4.4-12)	0.19	NA
C-X-C motif chemokine 6 (CXCL6)	CVD	6.4 (3.5-10.0)	1.40	NA
C-X-C motif chemokine 9 (CXCL9)	Onco	6.4 (0.4-11.3)	0.82	1
C-X-C motif chemokine-16 (CXCL16)	CVD	2.8 (0.9-4.8)	1.80	24
Cadherin-3 (CDH3)	Onco	1.5 (0.1-4.0)	0.11	12
Carbonic anhydrase IX (CAIX)	Onco	2 (0.3-7.4)	0.54	72
Caspase-3 (CASP3)	Onco	10.7 (5.6-13.5)	2.82	NA
Caspase-8 (CASP8)	CVD	1.5 (0.1-5.0)	0.20	62
Cathepsin D (CTSD)	CVD	6.8 (4.0-8.9)	3.30	NA
Cathepsin L1 (CTSL1)	CVD	5.5 (3.5-7.5)	1.0	NA
CD40 ligand (CD40L)	CVD/Onco	7.8 (3.4-11.7)	2.5	NA
Chitinase-3-like protein (CH3L1)	CVD	5.3 (1.6-10.1)	3.20	12
Cyclin-dependent kinase inhibitor 1A (CDKN1A)	Onco	2 (0.2-5.8)	0.34	114
Cystatin-B (CSTB)	CVD/Onco	4.7 (1.0-8.9)	1.90	1
Dickkopf-related protein-1 (Dkk1)	CVD	5.7 (3.2-8.7)	0.50	NA
E-selectin (SELE)	CVD/Onco	5.0 (2.6-11.0)	1.50	NA
Early activation antigen CD69 (CD60)	Onco	8.5 (0.5-11.5)	0.91	1
Endothelial cell-specific molecule 1 (ESM1)	CVD	2.9 (0.9-5.3)	0.60	NA
Eosinophil cationic protein (ECP)	CVD	5.0 (0.8-7.5)	1.60	2
Epidermal growth factor (EFG)	CVD	7.1 (2.4-10.6)	0.40	NA
Epidermal growth factor receptor (EGFR)	Onco	5.6 (2.1-7.2)	0.11	NA
Epididymal secretory protein E4 (HE4)	Onco	6.5 (3.6-8.6)	1.27	NA
Epithelial cell adhesion molecule (EpCAM)	Onco	9.9 (6.7-12.9)	1.54	NA
Eukaryotic translation initiation factor 4B (eIF4B)	Onco	6.5 (2.0-8.3)	1.84	NA

Extracellular matrix metalloproteinase inducer (EMMPRIN)	Onco	6.1 (0.03-7.1)	0.05	1
Ezrin (EZR)	Onco	4.1 (2.1-6.2)	1.42	NA
Fas antigen ligand (FasL)	Onco	1.3 (0.1-4.5)	0.28	74
FAS-associated death domain protein (FADD)	Onco	2.5 (1.1-5.9)	0.25	5
Fatty acid-binding protein, adipocyte (FABP4)	CVD	2.4 (0.7-5.2)	1.3	153
Fibroblast growth factor 23 (FGF23)	CVD	1.8 (0.0-6.9)	0.0	NA
Fms-related tyrosine kinase 3 ligand (Flt3L)	Onco	8.1 (5.5-10.0)	0.94	NA
Folate receptor alpha (FRalpha)	Onco	4.1 (1.0-7.1)	0.0	NA
Follistatin (FS)	CVD/Onco	4.8 (2.2-7.5)	1.7	NA
Fractalkine (CX3CL1)	CVD	5.0 (3.1-7.2)	2.3	NA
Furin (FUR)	Onco	6.5 (4.6-8.3)	0.43	NA
Galanin peptides (GAL)	CVD	5.0 (1.4-7.6)	0.9	NA
Galectin-3 (Gal3)	CVD	5.1 (3.0-7.1)	1.8	NA
Growth hormone (GH)	CVD/Onco	8.3 (2.8-12.3)	1.8	NA
Growth/differentiation factor 15 (GDF15)	CVD/Onco	8.7 (6.1-11.8)	2.6	NA
Heat shock 27 kDa protein (HSP27)	CVD	4.7 (0.6-6.7)	1.1	1
Heparin-binding EGF-like growth factor (HBEGF)	CVD/Onco	4.5 (2.9-6.5)	0.8	NA
Hepatocyte growth factor (HGF)	CVD/Onco	6.2 (3.9-10.1)	1.3	NA
ICOS ligand (ICOSLG)	Onco	3.2 (1.4-5.2)	1.23	NA
Immunoglobulin-like transcript 3 (ILT3)	Onco	1.8 (0.3-4.8)	0.55	17
Integrin alpha-1 (ITGA1)	Onco	6.3 (2.7-8.0)	0.63	NA
Interleukin-1 receptor antagonist protein (IL1ra)	CVD/Onco	3.9 (1.7-7.6)	3.4	616
Interleukin-12 (IL12)	Onco	7.6 (0.7-10.8)	1.44	1
Interleukin-16 (IL16)	CVD	3.7 (1.3-6.4)	0.0	NA
Interleukin-17 receptor B (IL17RB)	Onco	1.4 (0.1-4.6)	0.27	160
Interleukin-18 (IL18)	CVD	9.9 (6.7-12.7)	2.3	NA
Interleukin-27 subunit alpha (IL27A)	CVD	1.8 (0.2-3.3)	0.4	1
Interleukin-6 (IL6)	CVD/Onco	4.4 (1.8-12)	1.2	NA
Interleukin-6 receptor subunit alpha (IL6RA)	CVD/Onco	8.7 (6.6-10.3)	3.7	NA
Interleukin-7 (IL7)	Onco	1.3 (-0.1-5.5)	-0.06	55
Interleukin-8 (IL8)	CVD/Onco	4.7 (1.3-11.2)	2.5	2
Kallikrein-11 (hK11)	CVD/Onco	4.9 (1.2-8.9)	2.3	1
Kallikrein-6 (KLK6)	CVD/Onco	6.3 (3.8-8.8)	1.8	NA
Latency-associated peptide transforming growth factor beta-1 (LAPTGFbeta1)	Onco	5.3 (3.0-7.8)	0.53	NA
Lectin-like oxidized LDL receptor (LOX1)	CVD	4.1 (1.9-7.4)	1.3	NA
Leptin (LEP)	CVD	2.7 (0.1-5.4)	0.2	91
Macrophage colony-stimulating factor-1 (CSF1)	CVD/Onco	8.5 (6.1-10.2)	1.6	NA
Matrix metalloproteinase-1 (MMP1)	CVD/Onco	2.9 (0.1-7.7)	0.1	5
Matrix metalloproteinase-10 (MMP10)	CVD	6.0 (3.3-10.0)	1.3	NA
Matrix metalloproteinase-12 (MMP12)	CVD	6.6 (3.6-9.8)	0.6	NA
Matrix metalloproteinase-3 (MMP3)	CVD	0.2 (-1.8-4.4)	-0.9	81

Matrix metalloproteinase-7 (MMP7)	CVD	5.5 (1.2-8.4)	2.4	5
Melanoma-derived growth regulatory protein (MIA)	Onco	4.1 (0.5-5.9)	0.98	1
Melusin (ITGB1BP2)	CVD	4.2 (1.3-8.7)	2.6	169
Membrane-bound aminopeptidase P (mAmP)	CVD	2.3 (0.8-5.3)	1.5	1011
MHC class I polypeptide-related sequence A (MICA)	Onco	2.8 (0.2-5.4)	0.35	499
Midkine (MK)	Onco	6.3 (2.4-9.9)	0.71	NA
Monocyte chemotactic protein 1 (MCP1)	CVD/Onco	2.5 (0.5-6.5)	0.2	NA
Myeloid differentiation primary response protein MyD88 (MYD88)	Onco	1.1 (0.3-4.5)	0.6	1046
Myeloperoxidase (MPO)	CVD	3.5 (0.6-4.8)	1.2	2
Myoglobin (MB)	CVD	5.4 (3.1-8.1)	2.5	NA
N-terminal pro-B-type natriuretic peptide (NTproBNP)	CVD	3.2 (1.1-7.3)	2.2	746
NF-kappa-B essential modulator (NEMO)	CVD/Onco	5.0 (1.0-8.3)	2.0	2
NT-3 growth factor receptor (NTRK3)	Onco	6.4 (4.2-7.7)	0.81	NA
Osteoprotegerin (OPG)	CVD	9.3 (6.8-11.3)	0.8	NA
Ovarian cancer-related tumor marker CA 125 (CA125)	CVD/Onco	3.7 (1.4-7.3)	2.7	369
P-selectin glycoprotein ligand 1 (PSGL1)	CVD	0.5 (0.2-1.7)	0.3	1012
Pappalypsin-1 (PAPPA)	CVD	1.8 (0.5-5.2)	0.9	198
Parkinson protein 7 (PARK7)	Onco	7.5 (0.2-8.5)	0.38	1
Pentraxin-related protein PTX3 (PTX3)	CVD	1.2 (0.3-3.9)	0.5	245
Placenta growth factor (PlGF)	CVD/Onco	7 (4.6-9.1)	1.2	NA
Platelet endothelial cell adhesion molecule (PECAM1)	CVD/Onco	6.3 (4.2-8.3)	2.0	NA
Platelet-derived growth factor subunit B (PDGFsubunitB)	CVD/Onco	9.0 (5.4-11.2)	2.4	NA
Prolactin (PRL)	CVD/Onco	4.1 (0.7-7.5)	1.3	2
Prostasin (PRSS8)	Onco	8.3 (1.1-9.9)	2.27	1
Proteinase-activated receptor 1 (PAR1)	CVD	8.2 (5.9-9.6)	2.7	NA
Proto-oncogene tyrosine-protein kinase Src (SRC)	CVD	7.6 (4.0-9.9)	1.0	NA
Receptor for advanced glycosylation end products (RAGE)	CVD	4.3 (1.6-6.3)	0.2	NA
Receptor tyrosine-protein kinase erbB-2 (ErbB2HER2)	Onco	7.9 (0.5-9.4)	1.07	1
Receptor tyrosine-protein kinase erbB-3 (ErbB3HER3)	Onco	7.8 (5.7-9.0)	0.56	NA
Receptor tyrosine-protein kinase erbB-4 (ErbB4HER4)	Onco	4.7 (2.7-6.4)	-0.22	NA
Regenerating islet-derived protein 4 (REG4)	Onco	3.2 (0.7-5.5)	0.36	NA
Renin (REN)	CVD	6.5 (3.5-9.7)	1.5	NA
Resistin (RETN)	CVD	6.0 (3.4-10.1)	1.6	NA
SIR2-like protein-2 (SIRT2)	CVD	4.7 (0.5-8.4)	0.9	3
Spondin-1 (SPON1)	CVD	4.3 (1.9-6.6)	1.5	NA
ST2 protein (ST2)	CVD	2.7 (0.3 (5.5)	0.6	4
Stem cell factor (SCF)	CVD/Onco	6.8 (4.1-8.1)	0.0	NA
Tartrate-resistant acid phosphatase type 5 (TRAP)	Onco	5.2 (2.4-7.5)	0.27	NA
Thrombomodulin (TM)	CVD	9.3 (6.7-10.9)	2.1	NA

Thrombopoietin (THPO)	Onco	1.7 (0.6-5.8)	1.27	770
Tissue injury molecule-1/KIM-1 (TIM)	CVD	5.1 (1.1-9.6)	2.1	3
Tissue factor (TF)	CVD/Onco	5.6 (3.4-8.0)	0.9	NA
Tissue-type plasminogen activator (tPA)	CVD	5.3 (2.4-8.8)	1.5	NA
TNF-related activation-induced cytokine (TRANCE)	CVD	3.9 (1.2-6.2)	2.3	38
TNF-related apoptosis-inducing ligand (TRAIL)	CVD	8.3 (5.9-10.7)	1.4	NA
TNF-related apoptosis-inducing ligand receptor 2 (TRAILR2)	CVD/Onco	1.3 (-0.3-8.9)	-0.3	NA
Transforming growth factor alpha (TGFalpha)	Onco	1.3 (0.1-4.2)	0.17	3
Tumor necrosis factor ligand superfamily member 14 (TNFSF14)	CVD/Onco	2.2 (0.1-5.3)	0.2	2
Tumor necrosis factor receptor 1 (TNFR1)	CVD/Onco	12.2 (9.7-14.1)	1.7	NA
Tumor necrosis factor receptor 2 (TNFR2)	CVD/Onco	4.9 (2.3-7.0)	-0.1	NA
Tumor necrosis factor receptor superfamily member 4 (TNFRSF4)	Onco	2.0 (0.5-5.9)	0.2	NA
Tumor necrosis factor receptor superfamily member 5 (CD40)	CVD	9.4 (7.5-12.2)	1.4	NA
Tumor necrosis factor receptor superfamily member 6 (FAS)	CVD/Onco	7.3 (4.9-11.2)	0.9	NA
Tyrosine-protein kinase Lyn (LYN)	Onco	2.5 (0.2-4.2)	0.33	17
Tyrosine-protein phosphatase, non-receptor type 22 (PTPN22)	Onco	1.4 (0.6-5.4)	1.15	1336
Urokinase plasminogen activator surface receptor (UPAR)	CVD/Onco	9.5 (7.5-11.3)	1.3	NA
Vascular endothelial growth factor A (VEGFA)	CVD/Onco	10.0 (7.5-12.6)	1.1	NA
Vascular endothelial growth factor D (VEGFD)	CVD/Onco	6.7 (0.7-8.5)	1.3	2
Vascular endothelial growth factor receptor 2 (VEGFR2)	Onco	6.8 (4.4-8.0)	0.46	NA
Vimentin (VIM)	Onco	2.8 (0.4-6.5)	0.07	NA

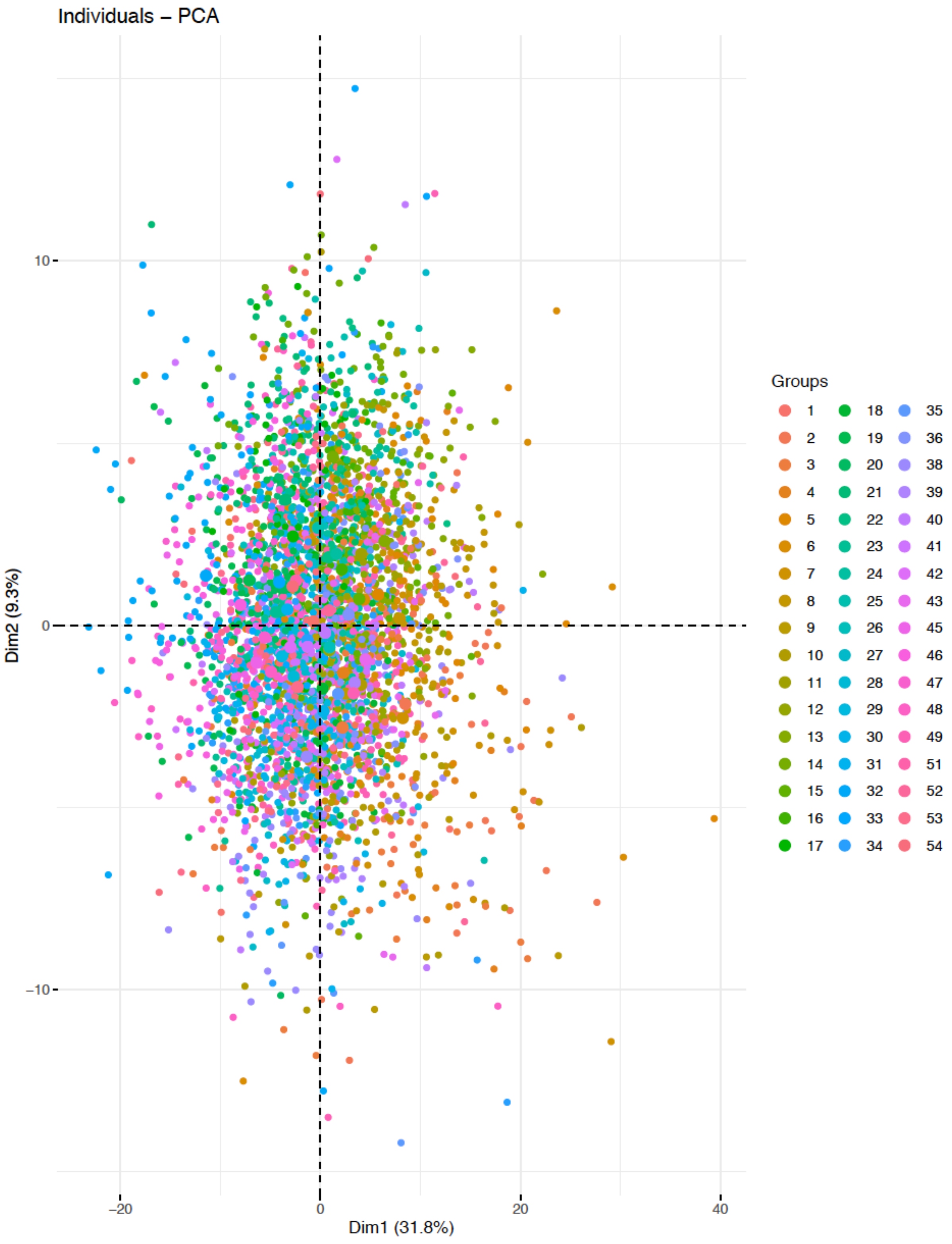

Supplement Figure 2. Principal components analysis (PCA) of 138 age-standardized plasma proteins in the MDCS-CVA (N=3,918). The first two principal components were plotted with each sample (i.e. individual) colored by batch group (i.e. plate).

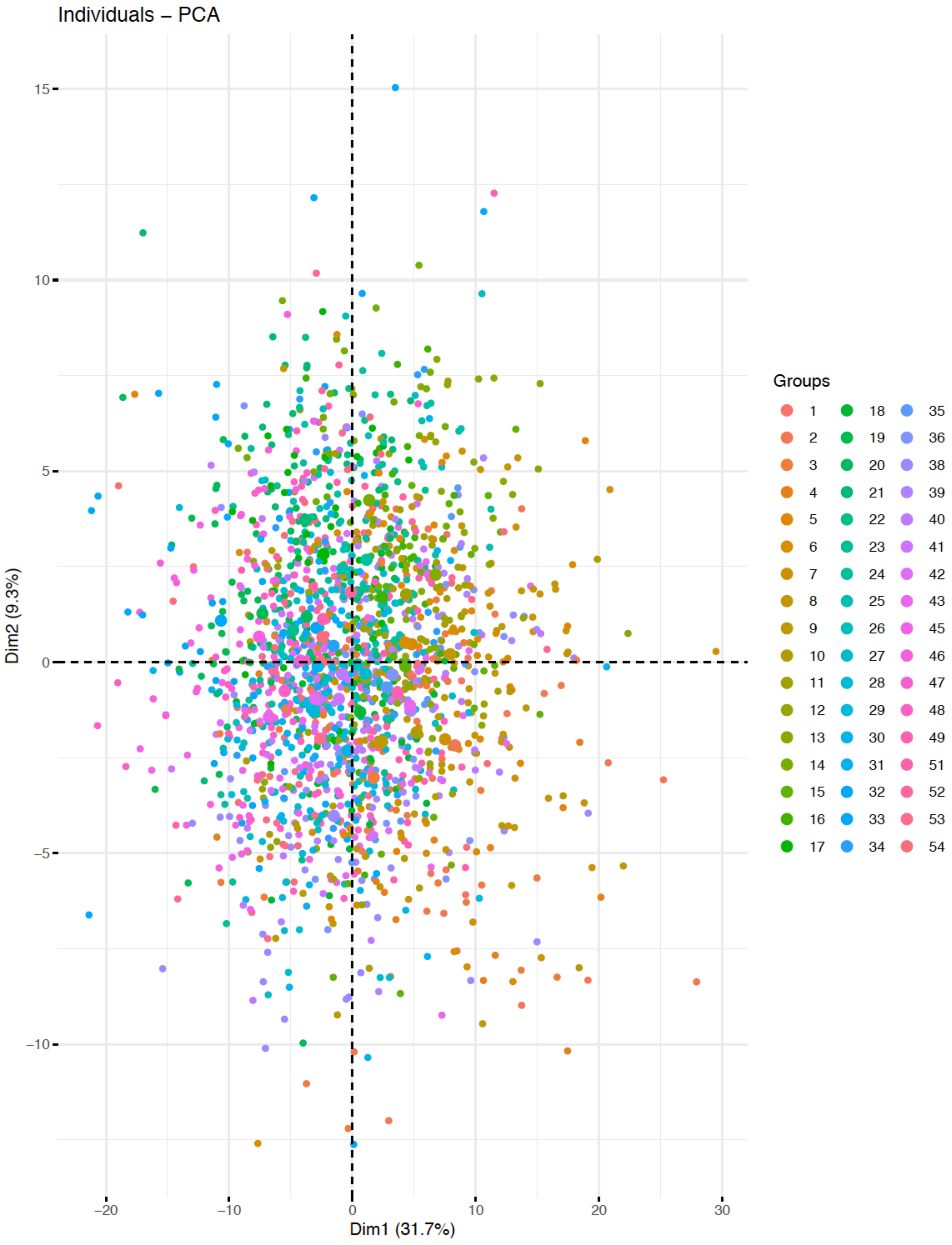

Supplement Figure 3. Principal components analysis (PCA) of 138 age-standardized plasma proteins in the MDCS-CVA random sample 1 (N=1,963). The first two principal components were plotted with each sample (i.e. individual) colored by batch group (i.e. plate).

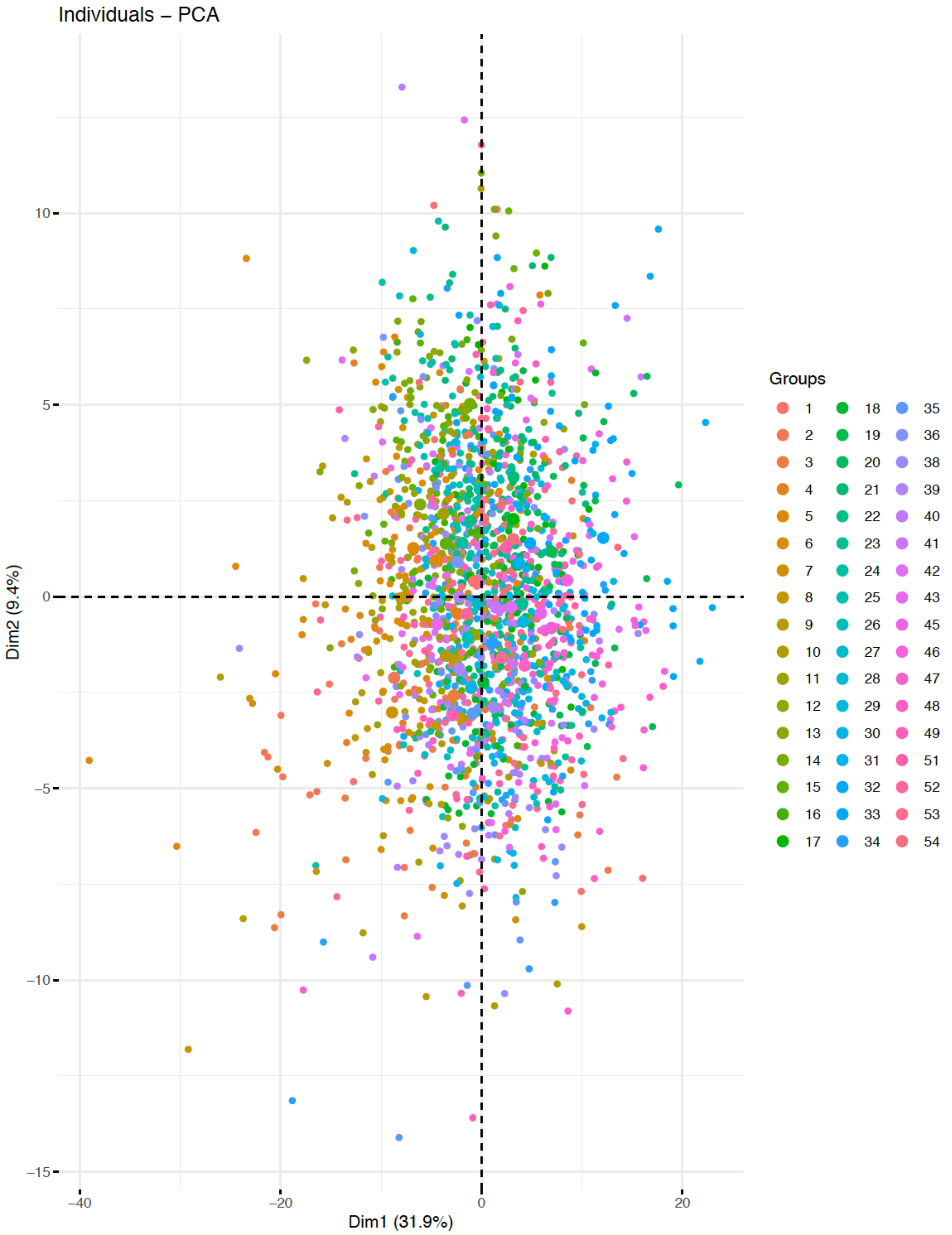

Supplement Figure 4. Principal components analysis (PCA) of 138 age-standardized plasma proteins in the MDCS-CVA random sample 2 (N=1,955). The first two principal components were plotted with each sample (i.e. individual) colored by batch group (i.e. plate).

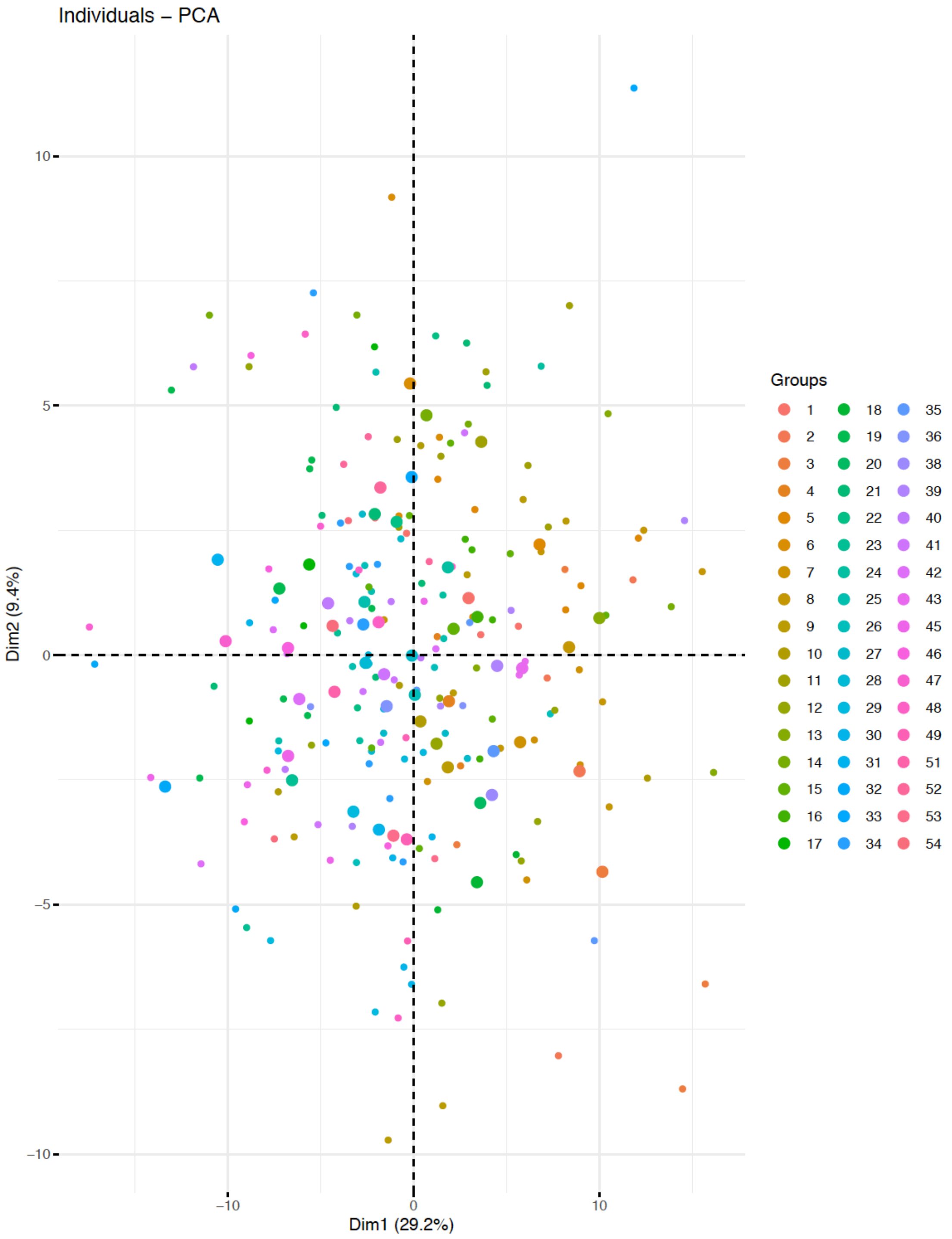

Supplement Figure 5. Principal components analysis (PCA) of 138 age-standardized plasma proteins in the MDCS-CVA in a smaller random sample (N=200). The first two principal components were plotted with each sample (i.e. individual) colored by batch group (i.e. plate).

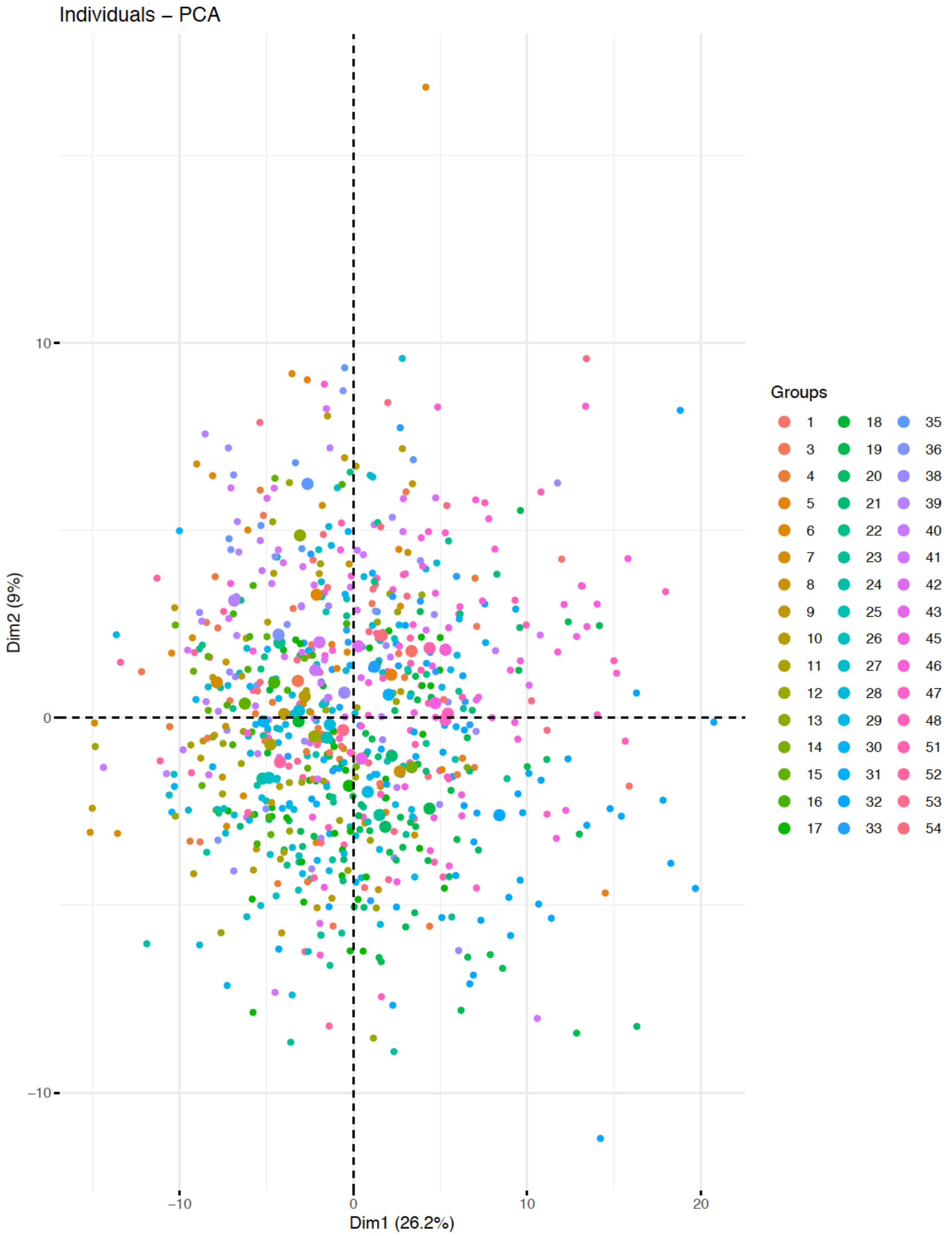

Supplement Figure 6. Principal components analysis (PCA) of 138 age-standardized plasma proteins in the MDCS-CVA among subjects with no imputed protein levels (N=772). The first two principal components were plotted with each sample (i.e. individual) colored by batch group (i.e. plate).

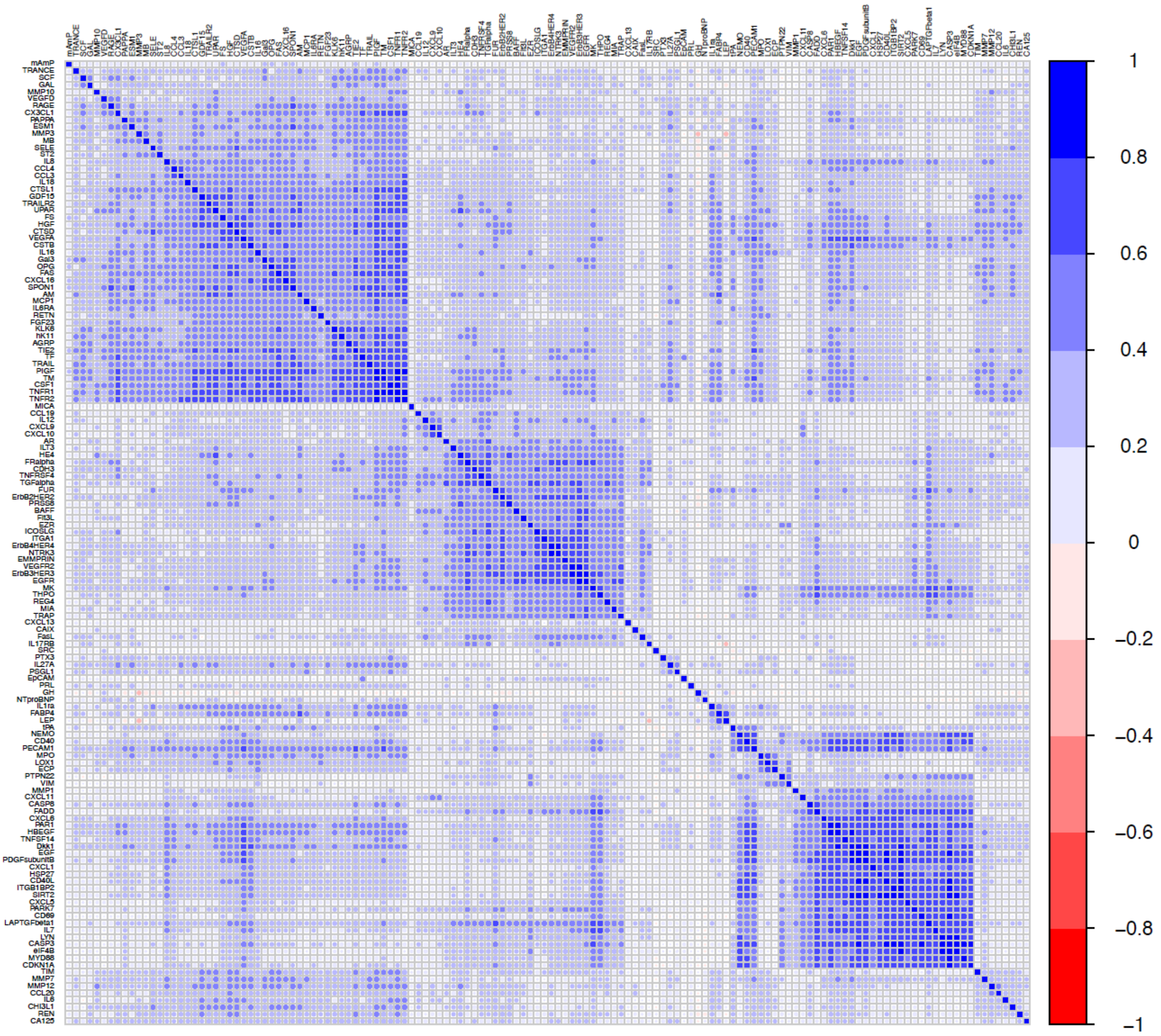

Supplement Figure 7. Heat map of Pearson correlation coefficients for 138 age-standardized plasma proteins levels measured in 3,918 subjects from the MDCS-CVA.

Supplement Table 2. Univariable and multivariable hazard ratios (HRs) and 95% confidence intervals (CI) per standard deviation (SD) of 138 proteins in relation to all-cause mortality in the MDCS-CVA (n=3,918). Multivariable analysis adjusted for age, sex, smoking status, BMI, educational level, history of hypertension, prevalent diabetes mellitus, C-reactive protein (ln-transformed), HbA1c (ln-transformed), and LDL-cholesterol.

Protein name (abbreviation)	Panel	Univariable	P-value	Multivariable	P-value
Adrenomedullin (AM)	CVD/Onco	1.09 (1.02-1.16)	0.012	1.01 (0.95-1.09)	0.678
Agouti-related protein (AGRP)	CVD	1.02 (0.96-1.08)	0.563	1.00 (0.94-1.07)	0.998
Amphiregulin (AR)	Onco	1.14 (1.08-1.20)	2.00E-06	1.13 (1.07-1.20)	3.60E-05
Angiopoietin-1 receptor (TIE2)	CVD/Onco	1.03 (0.97-1.10)	0.328	0.96 (0.90-1.03)	0.246
B-cell activating factor (BAFF)	Onco	1.07 (1.00-1.14)	0.036	1.01 (0.95-1.08)	0.688
C-C motif chemokine 19 (CCL19)	Onco	1.14 (1.08-1.21)	1.40E-05	1.09 (1.02-1.16)	7.70E-03
C-C motif chemokine 20 (CCL20)	CVD	1.14 (1.08-1.21)	6.60E-06	1.06 (1.00-1.13)	0.066
C-C motif chemokine 3 (CCL3)	CVD	1.11 (1.05-1.17)	3.00E-04	1.05 (0.99-1.12)	0.112
C-C motif chemokine 4 (CCL4)	CVD	1.06 (0.99-1.12)	0.079	0.98 (0.92-1.05)	0.643
C-X-C motif chemokine 1 (CXCL1)	CVD	1.06 (0.99-1.13)	0.094	0.99 (0.93-1.06)	0.874
C-X-C motif chemokine 10 (CXCL10)	Onco	1.05 (0.99-1.12)	0.092	1.09 (1.02-1.16)	0.012
C-X-C motif chemokine 11 (CXCL11)	Onco	1.06 (1.00-1.13)	0.053	1.09 (1.02-1.16)	7.20E-03
C-X-C motif chemokine 13 (CXCL13)	Onco	1.10 (1.02-1.18)	0.019	1.08 (1.01-1.16)	0.024
C-X-C motif chemokine 5 (CXCL5)	Onco	1.01 (0.95-1.08)	0.715	0.97 (0.91-1.04)	0.419
C-X-C motif chemokine 6 (CXCL6)	CVD	0.98 (0.92-1.05)	0.616	0.94 (0.88-1.00)	0.071
C-X-C motif chemokine 9 (CXCL9)	Onco	1.10 (1.04-1.17)	1.70E-03	1.13 (1.07-1.21)	8.90E-05
C-X-C motif chemokine-16 8CXCL16)	CVD	1.06 (1.00-1.13)	0.057	1.01 (0.95-1.07)	0.803
Cadherin-3 (CDH3)	Onco	0.98 (0.92-1.04)	0.527	0.97 (0.91-1.04)	0.419
Carbonic anhydrase IX (CAIX)	Onco	1.03 (0.97-1.09)	0.401	1.03 (0.97-1.10)	0.303
Caspase-3 (CASP3)	Onco	1.08 (1.01-1.15)	0.019	1.01 (0.94-1.07)	0.865
Caspase-8 (CASP8)	CVD	1.11 (1.04-1.18)	1.20E-03	1.02 (0.96-1.08)	0.593
Cathepsin D (CTSD)	CVD	1.23 (1.15-1.31)	1.70E-10	1.04 (0.97-1.11)	0.32
Cathepsin L1 (CTSL1)	CVD	1.17 (1.10-1.25)	1.1E-06	1.05 (0.98-1.12)	0.14
CD40 ligand (CD40L)	CVD/Onco	1.02 (0.96-1.09)	0.56	0.96 (0.90-1.02)	0.182
Chitinase-3-like protein (CH3L1)	CVD	1.20 (1.12-1.27)	1.40E-08	1.07 (1.00-1.13)	0.041
Cyclin-dependent kinase inhibitor 1A (CDKN1A)	Onco	1.06 (1.00-1.13)	0.062	0.98 (0.92-1.05)	0.612
Cystatin-B (CSTB)	CVD/Onco	1.17 (1.10-1.25)	1.00E-06	1.07 (1.00-1.14)	0.52
Dickkopf-related protein-1 (Dkk1)	CVD	0.99 (0.93-1.06)	0.782	0.95 (0.89-1.01)	0.125
E-selectin (SELE)	CVD/Onco	1.11 (1.05-1.19)	7.30E-04	0.98 (0.91-1.05)	0.496
Early activation antigen CD69 (CD60)	Onco	1.08 (1.02-1.16)	0.014	1.03 (0.96-1.10)	0.42
Endothelial cell-specific molecule 1 (ESM1)	CVD	0.92 (0.86-0.98)	9.50E-03	0.98 (0.92-1.04)	0.463
Eosinophil cationic protein (ECP)	CVD	1.06 (0.99-1.12)	0.085	1.01 (0.94-1.08)	0.823
Epidermal growth factor (EFG)	CVD	1.04 (0.97-1.11)	0.253	0.98 (0.92-1.04)	0.462
Epidermal growth factor receptor (EGFR)	Onco	0.92 (0.87-0.98)	0.011	0.89 (0.84-0.95)	4.90E-04

Epididymal secretory protein E4 (HE4)	Onco	1.35 (1.26-1.44)	6.60E-20	1.18 (1.09-1.27)	1.30E-05
Epithelial cell adhesion molecule (EpCAM)	Onco	0.99 (0.93-1.05)	0.711	1.02 (0.95-1.09)	0.571
Eukaryotic translation initiation factor 4B (eIF4B)	Onco	1.02 (0.96-1.09)	0.407	0.98 (0.92-1.04)	0.503
Extracellular matrix metalloproteinase inducer (EMMPRIN)	Onco	0.95 (0.89-1.00)	0.065	0.97 (0.92-1.03)	0.326
Ezrin (EZR)	Onco	1.00 (0.94-1.07)	0.884	0.97 (0.91-1.03)	0.319
Fas antigen ligand (FasL)	Onco	0.82 (0.77-0.88)	2.00E-09	0.92 (0.86-0.98)	0.012
FAS-associated death domain protein (FADD)	Onco	1.10 (1.03-1.17)	2.50E-03	1.03 (0.97-1.10)	0.373
Fatty acid-binding protein, adipocyte (FABP4)	CVD	0.96 (0.90-1.02)	0.166	0.97 (0.90-1.05)	0.478
Fibroblast growth factor 23 (FGF23)	CVD	1.12 (1.05-1.19)	3.30E-04	1.07 (1.00-1.14)	0.039
Fms-related tyrosine kinase 3 ligand (Flt3L)	Onco	1.04 (0.97-1.11)	0.272	1.00 (0.94-1.07)	0.973
Folate receptor alpha (FRalpha)	Onco	1.03 (0.96-1.09)	0.435	1.02 (0.96-1.09)	0.484
Follistatin (FS)	CVD/Onco	1.10 (1.04-1.17)	1.90E-03	0.97 (0.91-1.04)	0.454
Fractalkine (CX3CL1)	CVD	1.02 (0.96-1.09)	0.559	1.06 (0.99-1.12)	0.198
Furin (FUR)	Onco	1.14 (1.07-1.21)	5.50E-05	1.03 (0.95-1.11)	0.479
Galanin peptides (GAL)	CVD	0.94 (0.88-1.00)	0.049	0.92 (0.86-0.98)	0.013
Galectin-3 (Gal3)	CVD	0.93 (0.87-0.99)	0.026	0.95 (0.89-1.01)	0.111
Growth hormone (GH)	CVD/Onco	0.96 (0.90-1.02)	0.17	1.16 (1.08-1.26)	1.80E-04
Growth/differentiation factor 15 (GDF15)	CVD/Onco	1.42 (1.34-1.51)	2.40E-30	1.24 (1.16-1.33)	3.80E-10
Heat shock 27 kDa protein (HSP27)	CVD	0.99 (0.93-1.05)	0.645	0.96 (0.90-1.02)	0.158
Heparin-binding EGF-like growth factor (HBEGF)	CVD/Onco	1.01 (0.95-1.07)	0.798	0.96 (0.90-1.02)	0.196
Hepatocyte growth factor (HGF)	CVD/Onco	1.26 (1.18-1.34)	1.50E-12	1.08 (1.01-1.16)	0.31
ICOS ligand (ICOSLG)	Onco	0.92 (0.87-0.98)	0.013	0.94 (0.88-1.00)	0.04
Immunoglobulin-like transcript 3 (ILT3)	Onco	1.10 (1.04-1.17)	1.90E-03	1.05 (0.99-1.12)	0.095
Integrin alpha-1 (ITGA1)	Onco	0.98 (0.92-1.04)	0.497	1.00 (0.94-1.07)	0.997
Interleukin-1 receptor antagonist protein (IL1ra)	CVD/Onco	1.12 (1.05-1.19)	9.00E-04	1.03 (0.96-1.11)	0.426
Interleukin-12 (IL12)	Onco	0.99 (0.93-1.06)	0.87	1.08 (1.01-1.16)	0.021
Interleukin-16 (IL16)	CVD	1.03 (0.96-1.09)	0.425	0.96 (0.90-1.03)	0.241
Interleukin-17 receptor B (IL17RB)	Onco	0.99 (0.93-1.06)	0.77	0.98 (0.92-1.04)	0.499
Interleukin-18 (IL18)	CVD	1.13 (1.06-1.20)	1.00E-04	0.98 (0.92-1.05)	0.644
Interleukin-27 subunit alpha (IL27A)	CVD	1.01 (0.94-1.07)	0.837	1.04 (0.98-1.11)	0.2
Interleukin-6 (IL6)	CVD/Onco	1.23 (1.17-1.30)	2.40E-15	1.13 (1.05-1.20)	3.90E-04
Interleukin-6 receptor subunit alpha (IL6RA)	CVD/Onco	1.06 (0.99-1.13)	0.083	1.02 (0.96-1.09)	0.544
Interleukin-7 (IL7)	Onco	1.04 (0.98-1.11)	0.177	0.99 (0.93-1.06)	0.795
Interleukin-8 (IL8)	CVD/Onco	1.12 (1.06-1.19)	7.50E-05	1.06 (1.00-1.12)	0.071
Kallikrein-11 (hK11)	CVD/Onco	1.01 (0.95-1.08)	0.758	0.97 (0.91-1.03)	0.346
Kallikrein-6 (KLK6)	CVD/Onco	0.96 (0.90-1.02)	0.157	0.94 (0.88-1.00)	0.059
Latency-associated peptide transforming growth factor beta-1 (LAPTGFbeta1)	Onco	1.09 (1.02-1.16)	7.40E-03	1.02 (0.95-1.08)	0.64
Lectin-like oxidized LDL receptor (LOX1)	CVD	1.20 (1.13-1.28)	1.00E-09	1.11 (1.04-1.18)	2.20E-03
Leptin (LEP)	CVD	0.88 (0.83-0.94)	7.30E-05	0.95 (0.85-1.05)	0.305

Macrophage colony-stimulating factor-1 (CSF1)	CVD/Onco	1.11 (1.04-1.18)	1.40E-03	1.04 (0.97-1.11)	0.264
Matrix metalloproteinase-1 (MMP1)	CVD/Onco	1.15 (1.008-1.22)	1.60E-05	1.08 (1.01-1.15)	0.021
Matrix metalloproteinase-10 (MMP10)	CVD	1.06 (1.00-1.13)	0.05	0.99 (0.92-1.05)	0.686
Matrix metalloproteinase-12 (MMP12)	CVD	1.27 (1.19-1.35)	9.80E-14	1.10 (1.02-1.17)	8.90E-03
Matrix metalloproteinase-3 (MMP3)	CVD	1.21 (1.14-1.29)	1.30E-09	1.06 (0.99-1.15)	0.105
Matrix metalloproteinase-7 (MMP7)	CVD	1.15 (1.08-1.23)	3.20E-05	1.08 (1.01-1.16)	0.025
Melanoma-derived growth regulatory protein (MIA)	Onco	0.99 (0.93-1.05)	0.727	0.99 (0.93-1.06)	0.788
Melusin (ITGB1BP2)	CVD	1.06 (0.99-1.13)	0.081	1.00 (0.94-1.07)	0.904
Membrane-bound aminopeptidase P (mAmP)	CVD	0.98 (0.93-1.05)	0.635	0.99 (0.93-1.06)	0.846
MHC class I polypeptide-related sequence A (MICA)	Onco	0.98 (0.92-1.04)	0.547	0.96 (0.90-1.02)	0.203
Midkine (MK)	Onco	1.11 (1.04-1.18)	2.10E-03	1.05 (0.99-1.13)	0.124
Monocyte chemotactic protein 1 (MCP1)	CVD/Onco	1.04 (0.98-1.11)	0.168	1.00 (0.93-1.06)	0.933
Myeloid differentiation primary response protein MyD88 (MYD88)	Onco	1.03 (0.97-1.10)	0.287	0.99 (0.93-1.05)	0.703
Myeloperoxidase (MPO)	CVD	1.09 (1.02-1.16)	0.01	1.03 (0.96-1.10)	0.395
Myoglobin (MB)	CVD	0.99 (0.93-1.05)	0.702	0.91 (0.85-0.98)	8.30E-03
N-terminal pro-B-type natriuretic peptide (NTproBNP)	CVD	1.06 (0.99-1.13)	0.084	1.15 (1.08-1.23)	1.90E-05
NF-kappa-B essential modulator (NEMO)	CVD/Onco	1.00 (0.94-1.07)	0.882	0.96 (0.90-1.03)	0.245
NT-3 growth factor receptor (NTRK3)	Onco	0.92 (0.86-0.98)	8.90E-03	0.95 (0.89-1.01)	0.103
Osteoprotegerin (OPG)	CVD	1.11 (1.04-1.18)	1.60E-03	1.04 (0.98-1.11)	0.208
Ovarian cancer-related tumor marker CA 125 (CA125)	CVD/Onco	1.08 (1.01-1.15)	0.022	1.03 (0.97-1.10)	0.365
P-selectin glycoprotein ligand 1 (PSGL1)	CVD	0.99 (0.93-1.05)	0.727	0.98 (0.92-1.04)	0.513
Pappalypsin-1 (PAPPA)	CVD	1.08 (1.01-1.15)	0.023	1.02 (0.95-1.09)	0.651
Parkinson protein 7 (PARK7)	Onco	1.05 (0.99-1.13)	0.113	1.00 (0.94-1.06)	0.877
Pentraxin-related protein PTX3 (PTX3)	CVD	1.04 (0.98-1.11)	0.206	1.02 (0.96-1.09)	0.513
Placenta growth factor (PlGF)	CVD/Onco	1.16 (1.08-1.23)	1.10E-05	1.03 (0.97-1.11)	0.317
Platelet endothelial cell adhesion molecule (PECAM1)	CVD/Onco	1.04 (0.97-1.10)	0.257	0.97 (0.91-1.03)	0.33
Platelet-derived growth factor subunit B (PDGFsubunitB)	CVD/Onco	1.07 (1.00-1.14)	0.041	0.98 (0.92-1.05)	0.604
Prolactin (PRL)	CVD/Onco	0.95 (0.90-1.02)	0.145	1.00 (0.94-1.07)	0.918
Prostasin (PRSS8)	Onco	1.32 (1.23-1.41)	1.00E-15	1.11 (1.03-1.19)	7.00E-03
Proteinase-activated receptor 1 (PAR1)	CVD	1.01 (0.95-1.08)	0.667	0.96 (0.90-1.02)	0.169
Proto-oncogene tyrosine-protein kinase Src (SRC)	CVD	0.95 (0.89-1.01)	0.073	0.95 (0.90-1.01)	0.121
Receptor for advanced glycosylation end products (RAGE)	CVD	0.88 (0.83-0.94)	1.20E-04	0.93 (0.88-1.00)	0.035
Receptor tyrosine-protein kinase erbB-2 (ErbB2HER2)	Onco	1.08 (1.02-1.16)	0.016	0.97 (0.91-1.03)	0.368
Receptor tyrosine-protein kinase erbB-3 (ErbB3HER3)	Onco	0.97 (0.91-1.03)	0.328	0.95 (0.89-1.02)	0.152
Receptor tyrosine-protein kinase erbB-4 (ErbB4HER4)	Onco	1.01 (0.94-1.07)	0.877	0.95 (0.89-1.02)	0.152
Regenerating islet-derived protein 4 (REG4)	Onco	1.10 (1.03-1.17)	3.90E-03	1.03 (0.97-1.10)	0.302
Renin (REN)	CVD	1.22 (1.15-1.30)	4.90E-10	1.02 (0.96-1.09)	0.461
Resistin (RETN)	CVD	1.09 (1.02-1.16)	9.90E-03	1.04 (0.97-1.10)	0.267

SIR2-like protein-2 (SIRT2)	CVD	1.03 (0.96-1.09)	0.445	0.98 (0.91-1.04)	0.457
Spondin-1 (SPON1)	CVD	1.07 (1.00-1.14)	0.036	1.03 (0.96-1.10)	0.392
ST2 protein (ST2)	CVD	1.15 (1.08-1.22)	1.50E-05	1.07 (1.00-1.15)	0.055
Stem cell factor (SCF)	CVD/Onco	0.80 (0.75-0.85)	2.80E-14	0.86 (0.81-0.92)	6.20E-06
Tartrate-resistant acid phosphatase type 5 (TRAP)	Onco	1.09 (1.03-1.16)	6.40E-03	1.02 (0.96-1.09)	0.539
Thrombomodulin (TM)	CVD	1.05 (0.98-1.12)	0.142	0.97 (0.91-1.03)	0.323
Thrombopoietin (THPO)	Onco	0.99 (0.93-1.06)	0.78	0.95 (0.89-1.02)	0.153
Tissue injury molecule-1/KIM-1 (TIM)	CVD	1.26 (1.18-1.34)	8.00E-13	1.13 (1.05-1.20)	4.40E-04
Tissue factor (TF)	CVD/Onco	0.99 (0.93-1.05)	0.712	0.97 (0.91-1.04)	0.399
Tissue-type plasminogen activator (tPA)	CVD	1.09 (1.02-1.16)	8.10E-03	1.00 (0.94-1.07)	0.956
TNF-related activation-induced cytokine (TRANCE)	CVD	0.97 (0.91-1.03)	0.316	0.94 (0.88-1.00)	0.065
TNF-related apoptosis-inducing ligand (TRAIL)	CVD	1.00 (0.94-1.07)	0.907	0.92 (0.86-0.98)	0.013
TNF-related apoptosis-inducing ligand receptor 2 (TRAILR2)	CVD/Onco	1.14 (1.10-1.18)	1.50E-11	1.07 (1.02-1.13)	8.40E-03
Transforming growth factor alpha (TGFalpha)	Onco	1.13 (1.07-1.21)	7.60E-05	1.08 (1.01-1.15)	0.023
Tumor necrosis factor ligand superfamily member 14 (TNFSF14)	CVD/Onco	1.07 (1.01-1.14)	0.027	0.99 (0.93-1.05)	0.725
Tumor necrosis factor receptor 1 (TNFR1)	CVD/Onco	1.15 (1.07-1.22)	4.00E-05	1.03 (0.96-1.10)	0.387
Tumor necrosis factor receptor 2 (TNFR2)	CVD/Onco	1.14 (1.07-1.22)	3.4E-06	1.03 (0.97-1.11)	0.23
Tumor necrosis factor receptor superfamily member 4 (TNFRSF4)	Onco	1.09 (1.02-1.16)	7.30E-03	1.04 (0.97-1.11)	0.253
Tumor necrosis factor receptor superfamily member 5 (CD40)	CVD	1.04 (0.98-1.11)	0.242	0.99 (0.93-1.06)	0.834
Tumor necrosis factor receptor superfamily member 6 (FAS)	CVD/Onco	1.07 (1.01-1.14)	0.029	0.97 (0.91-1.04)	0.424
Tyrosine-protein kinase Lyn (LYN)	Onco	1.03 (0.97-1.10)	0.379	0.98 (0.92-1.04)	0.494
Tyrosine-protein phosphatase, non-receptor type 22 (PTPN22)	Onco	1.10 (1.03-1.17)	2.20E-03	1.03 (0.97-1.10)	0.299
Urokinase plasminogen activator surface receptor (UPAR)	CVD/Onco	1.29 (1.21-1.37)	6.90E-15	1.16 (1.08-1.24)	4.70E-05
Vascular endothelial growth factor A (VEGFA)	CVD/Onco	1.13 (1.06-1.20)	7.90E-05	1.03 (0.96-1.10)	0.44
Vascular endothelial growth factor D (VEGFD)	CVD/Onco	1.01 (0.94-1.08)	0.81	0.99 (0.93-1.06)	0.799
Vascular endothelial growth factor receptor 2 (VEGFR2)	Onco	0.98 (0.92-1.04)	0.539	0.93 (0.87-0.99)	0.02
Vimentin (VIM)	Onco	1.03 (0.97-1.10)	0.317	1.00 (0.94-1.07)	0.992

Supplement Table 3. Multivariable hazard ratios (HR) and 95% confidence intervals (CI) per standard deviation (SD) for 138 proteins in relation to all-cause mortality in two random samples of the MDCS-CVA (n=3,918). Multivariable analyses adjusted for age, sex, smoking status, BMI, educational level, history of hypertension, prevalent diabetes mellitus, C-reactive protein (ln-transformed), HbA1c (ln-transformed), and LDL-cholesterol. Proteins with a nominally significant association with all-cause mortality (P<0.05) in one (light grey) or both (dark grey) random samples are highlighted.

**Protein name (abbreviation)**	**Panel**	**Random sample 1**	**P-value**	**Random sample 2**	**P-value**
Adrenomedullin (AM)	CVD/Onco	1.10 (1.00-1.21)	0.063	0.93 (0.84-1.02)	0.131
Agouti-related protein (AGRP)	CVD	1.03 (0.94-1.13)	0.581	0.97 (0.89-1.07)	0.585
Amphiregulin (AR)	Onco	1.14 (1.05-1.23)	1.00E-03	1.13 (1.03-1.24)	0.011
Angiopoietin-1 receptor (TIE2)	CVD/Onco	0.99 (0.90-1.09)	0.874	0.93 (0.84-1.02)	0.107
B-cell activating factor (BAFF)	Onco	1.04 (0.95-1.14)	0.385	0.97 (0.88-1.07)	0.575
C-C motif chemokine 19 (CCL19)	Onco	1.04 (0.95-1.14)	0.400	1.14 (1.04-1.25)	4.60E-03
C-C motif chemokine 20 (CCL20)	CVD	1.07 (0.98-1.17)	0.147	1.05 (0.96-1.15)	0.302
C-C motif chemokine 3 (CCL3)	CVD	1.07 (0.97-1.17)	0.161	1.03 (0.95-1.13)	0.463
C-C motif chemokine 4 (CCL4)	CVD	1.02 (0.93-1.12)	0.696	0.95 (0.87-1.04)	0.297
C-X-C motif chemokine 1 (CXCL1)	CVD	1.00 (0.91-1.10)	0.998	0.99 (0.90-1.09)	0.842
C-X-C motif chemokine 10 (CXCL10)	Onco	1.08 (0.99-1.19)	0.079	1.09 (0.99-1.19)	0.088
C-X-C motif chemokine 11 (CXCL11)	Onco	1.10 (1.01-1.20)	0.032	1.08 (0.98-1.18)	0.111
C-X-C motif chemokine 13 (CXCL13)	Onco	1.07 (0.97-1.18)	0.173	1.09 (0.99-1.21)	0.091
C-X-C motif chemokine 5 (CXCL5)	Onco	0.98 (0.89-1.08)	0.722	0.97 (0.88-1.06)	0.472
C-X-C motif chemokine 6 (CXCL6)	CVD	0.95 (0.87-1.04)	0.274	0.93 (0.85-1.02)	0.141
C-X-C motif chemokine 9 (CXCL9)	Onco	1.11 (1.01-1.22)	0.023	1.15 (1.05-1.26)	2.00E-03
C-X-C motif chemokine-16 8CXCL16)	CVD	1.03 (0.94-1.13)	0.56	0.99 (0.90-1.08)	0.811
Cadherin-3 (CDH3)	Onco	1.02 (0.93-1.11)	0.685	0.92 (0.84-1.01)	0.081
Carbonic anhydrase IX (CAIX)	Onco	1.04 (0.95-1.13)	0.417	1.03 (0.94-1.12)	0.582
Caspase-3 (CASP3)	Onco	1.00 (0.91-1.10)	0.97	1.01 (0.92-1.11)	0.814
Caspase-8 (CASP8)	CVD	1.04 (0.95-1.13)	0.392	0.99 (0.91-1.09)	0.882
Cathepsin D (CTSD)	CVD	1.05 (0.95-1.16)	0.307	1.02 (0.92-1.12)	0.754
Cathepsin L1 (CTSL1)	CVD	1.10 (1.00-1.20)	0.053	1.00 (0.91-1.10)	0.986
CD40 ligand (CD40L)	CVD/Onco	0.96 (0.87-1.05)	0.337	0.96 (0.88-1.05)	0.345
Chitinase-3-like protein (CH3L1)	CVD	1.11 (1.01-1.21)	0.025	1.02 (0.94-1.12)	0.61
Cyclin-dependent kinase inhibitor 1A (CDKN1A)	Onco	0.98 (0.89-1.07)	0.607	0.99 (0.90-1.08)	0.798
Cystatin-B (CSTB)	CVD/Onco	1.10 (1.00-1.21)	0.057	1.04 (0.94-1.14)	0.446
Dickkopf-related protein-1 (Dkk1)	CVD	0.97 (0.88-1.06)	0.522	0.93 (0.85-1.02)	0.12
E-selectin (SELE)	CVD/Onco	0.97 (0.88-1.07)	0.553	0.98 (0.89-1.08)	0.684
Early activation antigen CD69 (CD60)	Onco	1.03 (0.94-1.13)	0.508	1.02 (0.93-1.12)	0.642
Endothelial cell-specific molecule 1 (ESM1)	CVD	1.00 (0.92-1.10)	0.933	0.95 (0.86-1.04)	0.246
Eosinophil cationic protein (ECP)	CVD	1.06 (0.97-1.16)	0.194	0.95 (0.86-1.04)	0.259
Epidermal growth factor (EFG)	CVD	0.99 (0.90-1.08)	0.807	0.97 (0.89-1.06)	0.462

Epidermal growth factor receptor (EGFR)	Onco	0.93 (0.86-1.02)	0.13	0.84 (0.76-0.92)	2.60E-04
Epididymal secretory protein E4 (HE4)	Onco	1.22 (1.10-1.35)	1.70E-04	1.13 (1.02-1.26)	0.023
Epithelial cell adhesion molecule (EpCAM)	Onco	1.01 (0.92-1.11)	0.859	1.04 (0.94-1.14)	0.467
Eukaryotic translation initiation factor 4B (eIF4B)	Onco	0.95 (0.87-1.05)	0.338	1.00 (0.91-1.09)	0.986
Extracellular matrix metalloproteinase inducer (EMMPRIN)	Onco	0.97 (0.90-1.04)	0.412	0.97 (0.87-1.07)	0.527
Ezrin (EZR)	Onco	0.98 (0.90-1.08)	0.745	0.94 (0.86-1.04)	0.23
Fas antigen ligand (FasL)	Onco	0.95 (0.87-1.04)	0.263	0.87 (0.79-0.96)	7.00E-03
FAS-associated death domain protein (FADD)	Onco	1.07 (0.98-1.17)	0.152	0.99 (0.90-1.08)	0.821
Fatty acid-binding protein, adipocyte (FABP4)	CVD	0.99 (0.89-1.11)	0.904	0.95 (0.85-1.06)	0.339
Fibroblast growth factor 23 (FGF23)	CVD	1.13 (1.04-1.24)	5.30E-03	1.00 (0.91-1.10)	0.984
Fms-related tyrosine kinase 3 ligand (Flt3L)	Onco	1.01 (0.92-1.11)	0.814	0.98 (0.90-1.08)	0.695
Folate receptor alpha (FRalpha)	Onco	1.05 (0.96-1.15)	0.323	0.99 (0.90-1.09)	0.855
Follistatin (FS)	CVD/Onco	1.03 (0.93-1.13)	0.604	0.92 (0.83-1.01)	0.094
Fractalkine (CX3CL1)	CVD	1.07 (0.98-1.17)	0.121	1.00 (0.92-1.10)	0.939
Furin (FUR)	Onco	1.03 (0.93-1.14)	0.624	1.02 (0.92-1.14)	0.668
Galanin peptides (GAL)	CVD	0.92 (0.84-1.02)	0.112	0.91 (0.83-1.00)	0.055
Galectin-3 (Gal3)	CVD	0.97 (0.89-1.06)	0.521	0.92 (0.84-1.01)	0.087
Growth hormone (GH)	CVD/Onco	1.20 (1.07-1.34)	1.50E-03	1.13 (1.011.27)	0.035
Growth/differentiation factor 15 (GDF15)	CVD/Onco	1.26 (1.14-1.38)	3.00E-06	1.23 (1.11-1.35)	3.30E-05
Heat shock 27 kDa protein (HSP27)	CVD	0.95 (0.87-1.04)	0.293	0.96 (0.88-1.05)	0.36
Heparin-binding EGF-like growth factor (HBEGF)	CVD/Onco	0.96 (0.88-1.05)	0.407	0.95 (0.87-1.05)	0.322
Hepatocyte growth factor (HGF)	CVD/Onco	0.99 (0.90-1.08)	0.807	0.97 (0.89-1.06)	0.462
ICOS ligand (ICOSLG)	Onco	0.94 (0.86-1.03)	0.189	0.92 (0.84-1.01)	0.088
Immunoglobulin-like transcript 3 (ILT3)	Onco	1.08 (0.99-1.18)	0.09	1.03 (0.94-1.12)	0.546
Integrin alpha-1 (ITGA1)	Onco	1.06 (0.97-1.16)	0.21	0.93 (0.85-1.03)	0.148
Interleukin-1 receptor antagonist protein (IL1ra)	CVD/Onco	1.03 (0.92-1.15)	0.619	1.03 (0.93-1.14)	0.595
Interleukin-12 (IL12)	Onco	1.07 (0.97-1.17)	0.173	1.09 (0.99-1.21)	0.08
Interleukin-16 (IL16)	CVD	0.98 (0.90-1.08)	0.732	0.94 (0.85-1.03)	0.162
Interleukin-17 receptor B (IL17RB)	Onco	1.05 (0.96-1.15)	0.294	0.91 (0.83-1.00)	0.041
Interleukin-18 (IL18)	CVD	1.00 (0.91-1.10)	0.997	0.97 (0.88-1.07)	0.51
Interleukin-27 subunit alpha (IL27A)	CVD	1.06 (0.97-1.16)	0.219	1.03 (0.94-1.13)	0.511
Interleukin-6 (IL6)	CVD/Onco	1.14 (1.04-1.24)	4.20E-03	1.11 (1.00-1.22)	0.045
Interleukin-6 receptor subunit alpha (IL6RA)	CVD/Onco	1.04 (0.95-1.14)	0.419	1.00 (0.91-1.09)	0.96
Interleukin-7 (IL7)	Onco	1.01 (0.92-1.11)	0.812	0.97 (0.89-1.06)	0.509
Interleukin-8 (IL8)	CVD/Onco	1.07 (0.99-1.16)	0.091	1.04 (0.94-1.14)	0.443
Kallikrein-11 (hK11)	CVD/Onco	1.00 (0.91-1.09)	0.936	0.93 (0.85-1.02)	0.146
Kallikrein-6 (KLK6)	CVD/Onco	0.96 (0.88-1.05)	0.426	0.91 (0.83-1.00)	0.039
Latency-associated peptide transforming growth factor beta-1 (LAPTGFbeta1)	Onco	1.05 (0.96-1.15)	0.279	0.98 (0.89-1.07)	0.602
Lectin-like oxidized LDL receptor (LOX1)	CVD	1.13 (1.03-1.23)	9.20E-03	1.08 (0.99-1.19)	0.096
Leptin (LEP)	CVD	0.92 (0.79-1.07)	0.27	0.97 (0.83-1.12)	0.668

Macrophage colony-stimulating factor-1 (CSF1)	CVD/Onco	1.07 (0.97-1.18)	0.148	1.01 (0.92-1.11)	0.848
Matrix metalloproteinase-1 (MMP1)	CVD/Onco	1.08 (0.98-1.18)	0.105	1.08 (0.99-1.18)	0.075
Matrix metalloproteinase-10 (MMP10)	CVD	0.98 (0.89-1.08)	0.685	0.99 (0.90-1.09)	0.832
Matrix metalloproteinase-12 (MMP12)	CVD	1.10 (1.00-1.21)	0.058	1.08 (0.98-1.19)	0.108
Matrix metalloproteinase-3 (MMP3)	CVD	1.10 (0.99-1.22)	0.09	1.02 (0.92-1.14)	0.678
Matrix metalloproteinase-7 (MMP7)	CVD	1.09 (0.99-1.20)	0.064	1.07 (0.97-1.17)	0.187
Melanoma-derived growth regulatory protein (MIA)	Onco	1.01 (0.92-1.10)	0.847	0.97 (0.88-1.06)	0.5
Melusin (ITGB1BP2)	CVD	0.98 (0.90-1.08)	0.691	1.02 (0.94-1.12)	0.599
Membrane-bound aminopeptidase P (mAmP)	CVD	1.02 (0.94-1.12)	0.609	0.96 (0.88-1.05)	0.378
MHC class I polypeptide-related sequence A (MICA)	Onco	0.91 (0.84-0.99)	0.028	1.02 (0.93-1.12)	0.678
Midkine (MK)	Onco	1.09 (0.99-1.19)	0.088	1.02 (0.93-1.12)	0.71
Monocyte chemotactic protein 1 (MCP1)	CVD/Onco	1.06 (0.96-1.16)	0.231	0.94 (0.86-1.03)	0.17
Myeloid differentiation primary response protein MyD88 (MYD88)	Onco	0.98 (0.89-1.07)	0.638	1.00 (0.91-1.09)	0.919
Myeloperoxidase (MPO)	CVD	1.11 (1.02-1.22)	0.02	0.93 (0.85-1.03)	0.162
Myoglobin (MB)	CVD	0.93 (0.85-1.03)	0.15	0.88 (0.80-0.98)	0.014
N-terminal pro-B-type natriuretic peptide (NTproBNP)	CVD	1.17 (1.07-1.29)	4.60E-04	1.12 (1.02-1.23)	0.019
NF-kappa-B essential modulator (NEMO)	CVD/Onco	0.92 (0.84-1.01)	0.077	1.01 (0.92-1.10)	0.905
NT-3 growth factor receptor (NTRK3)	Onco	0.98 (0.90-1.08)	0.706	0.90 (0.82-0.99)	0.029
Osteoprotegerin (OPG)	CVD	1.07 (0.97-1.17)	0.182	1.02 (0.93-1.12)	0.739
Ovarian cancer-related tumor marker CA 125 (CA125)	CVD/Onco	1.10 (1.01-1.21)	0.037	0.96 (0.88-1.05)	0.404
P-selectin glycoprotein ligand 1 (PSGL1)	CVD	0.95 (0.86-1.04)	0.27	1.01 (0.92-1.11)	0.8
Pappalypsin-1 (PAPPA)	CVD	1.09 (0.99-1.20)	0.073	0.95 (0.86-1.04)	0.275
Parkinson protein 7 (PARK7)	Onco	1.00 (0.92-1.08)	0.963	0.99 (0.90-1.09)	0.866
Pentraxin-related protein PTX3 (PTX3)	CVD	1.08 (0.98-1.18)	0.104	0.97 (0.88-1.06)	0.517
Placenta growth factor (PlGF)	CVD/Onco	1.09 (0.99-1.20)	0.081	0.98 (0.89-1.08)	0.66
Platelet endothelial cell adhesion molecule (PECAM1)	CVD/Onco	0.96 (0.88-1.05)	0.394	0.97 (0.89-1.06)	0.509
Platelet-derived growth factor subunit B (PDGFsubunitB)	CVD/Onco	1.01 (0.92-1.11)	0.764	0.95 (0.87-1.04)	0.291
Prolactin (PRL)	CVD/Onco	1.08 (0.98-1.19)	0.111	0.93 (0.84-1.02)	0.123
Prostasin (PRSS8)	Onco	1.12 (1.02-1.24)	0.024	1.08 (0.97-1.20)	0.148
Proteinase-activated receptor 1 (PAR1)	CVD	0.97 (0.89-1.07)	0.552	0.94 (0.86-1.03)	0.181
Proto-oncogene tyrosine-protein kinase Src (SRC)	CVD	0.93 (0.84-1.02)	0.101	0.98 (0.90-1.06)	0.585
Receptor for advanced glycosylation end products (RAGE)	CVD	0.93 (0.85-1.01)	0.099	0.94 (0.86-1.02)	0.151
Receptor tyrosine-protein kinase erbB-2 (ErbB2HER2)	Onco	0.99 (0.91-1.08)	0.852	0.94 (0.85-1.04)	0.211
Receptor tyrosine-protein kinase erbB-3 (ErbB3HER3)	Onco	0.99 (0.90-1.09)	0.885	0.91 (0.83-1.00)	0.045
Receptor tyrosine-protein kinase erbB-4 (ErbB4HER4)	Onco	1.00 (0.91-1.10)	0.944	0.91 (0.83-1.00)	0.042
Regenerating islet-derived protein 4 (REG4)	Onco	1.09 (0.99-1.19)	0.087	0.99 (0.90-1.08)	0.771
Renin (REN)	CVD	1.04 (0.95-1.14)	0.422	1.01 (0.92-1.11)	0.89
Resistin (RETN)	CVD	1.04 (0.94-1.14)	0.44	1.02 (0.94-1.12)	0.609

SIR2-like protein-2 (SIRT2)	CVD	0.96 (0.88-1.05)	0.405	0.99 (0.90-1.08)	0.79
Spondin-1 (SPON1)	CVD	1.06 (0.97-1.16)	0.218	0.99 (0.91-1.09)	0.883
ST2 protein (ST2)	CVD	1.07 (0.97-1.18)	0.163	1.06 (0.96-1.18)	0.252
Stem cell factor (SCF)	CVD/Onco	0.88 (0.81-0.96)	5.80E-03	0.84 (0.76-0.92)	1.60E-04
Tartrate-resistant acid phosphatase type 5 (TRAP)	Onco	1.05 (0.95-1.15)	0.326	0.99 (0.90-1.09)	0.894
Thrombomodulin (TM)	CVD	1.01 (0.92-1.11)	0.784	0.91 (0.83-1.00)	0.058
Thrombopoietin (THPO)	Onco	0.97 (0.89-1.07)	0.579	0.93 (0.85-1.02)	0.145
Tissue injury molecule-1/KIM-1 (TIM)	CVD	1.17 (1.07-1.29)	9.60E-04	1.08 (0.98-1.19)	0.11
Tissue factor (TF)	CVD/Onco	0.99 (0.90-1.08)	0.759	0.95 (0.87-1.05)	0.302
Tissue-type plasminogen activator (tPA)	CVD	0.95 (0.87-1.05)	0.31	1.05 (0.96-1.16)	0.273
TNF-related activation-induced cytokine (TRANCE)	CVD	0.93 (0.85-1.02)	0.125	0.94 (0.86-1.04)	0.232
TNF-related apoptosis-inducing ligand (TRAIL)	CVD	0.93 (0.85-1.02)	0.117	0.91 (0.83-1.00)	0.04
TNF-related apoptosis-inducing ligand receptor 2 (TRAILR2)	CVD/Onco	1.14 (1.07-1.22)	1.00E-04	1.01 (0.92-1.10)	0.832
Transforming growth factor alpha (TGFalpha)	Onco	1.12 (1.03-1.22)	7.90E-03	1.02 (0.93-1.12)	0.641
Tumor necrosis factor ligand superfamily member 14 (TNFSF14)	CVD/Onco	1.03 (0.93-1.13)	0.589	0.96 (0.87-1.05)	0.332
Tumor necrosis factor receptor 1 (TNFR1)	CVD/Onco	1.04 (0.94-1.14)	0.452	1.01 (0.92-1.12)	0.784
Tumor necrosis factor receptor 2 (TNFR2)	CVD/Onco	1.07 (0.97-1.18)	0.156	1.01 (0.91-1.11)	0.906
Tumor necrosis factor receptor superfamily member 4 (TNFRSF4)	Onco	1.06 (0.97-1.16)	0.196	1.01 (0.92-1.11)	0.86
Tumor necrosis factor receptor superfamily member 5 (CD40)	CVD	0.97 (0.88-1.06)	0.467	1.02 (0.93-1.11)	0.712
Tumor necrosis factor receptor superfamily member 6 (FAS)	CVD/Onco	1.00 (0.91-1.10)	0.99	0.94 (0.86-1.03)	0.197
Tyrosine-protein kinase Lyn (LYN)	Onco	0.98 (0.89-1.07)	0.647	0.97 (0.89-1.06)	0.553
Tyrosine-protein phosphatase, non-receptor type 22 (PTPN22)	Onco	1.04 (0.95-1.14)	0.347	1.02 (0.93-1.12)	0.651
Urokinase plasminogen activator surface receptor (UPAR)	CVD/Onco	1.20 (1.08-1.32)	3.70E-04	1.12 (1.01-1.23)	0.031
Vascular endothelial growth factor A (VEGFA)	CVD/Onco	1.07 (0.97-1.17)	0.192	0.99 (0.90-1.08)	0.801
Vascular endothelial growth factor D (VEGFD)	CVD/Onco	0.97 (0.88-1.06)	0.477	1.01 (0.92-1.12)	0.769
Vascular endothelial growth factor receptor 2 (VEGFR2)	Onco	0.97 (0.88-1.07)	0.508	0.88 (0.80-0.96)	5.90E-03
Vimentin (VIM)	Onco	1.03 (0.94-1.13)	0.512	0.96 (0.87-1.06)	0.414

Supplement Table 4. Mean RSF-derived prediction error rates for all-cause mortality in the MDCS-CVA using in three RSF models in two random samples and the full study population.

RSF model	Mean prediction error rate (95% CI)		
	Random sample 1	Random sample 2	Full study population###
Only covariates*	0.3075 (0.3073-0.3078)	0.3114 (0.3111-0.3117)	0.3013 (0.3011-0.3016)
Covariates and selected proteins#	0.2683 (0.2679-0.2686)	0.2814 (0.2811-0.2818)	0.2717 (0.2715-0.2719)
Covariates and all proteins##	0.2853 (0.2848-0.2858)	0.3012 (0.3008-0.3017)	0.2799 (0.2796-0.2803)

* Covariates included age, sex, smoking status, BMI, educational level, history of hypertension, prevalent diabetes mellitus, C-reactive protein, HbA1c, and LDL-cholesterol.

# Selected proteins based on the random survival forest backward algorithm. In total, 49 and 30 proteins were retained in random sample 1 and 2, respectively.

RSF model including covariates and all 138 proteins. .

RSF models in full study population including covariates only, all proteins and a model including covariates and proteins retained using the RSF backward algorithm in both random samples (21 proteins; FABP4, FasL, GDF15, HE4, HGF, IL12, IL6, mAmP, MMP1, MMP12, MYD88, NTproBNP, PRSS8, PSGL1, PTPN22, PTX3, RAGE, REN, SCF, THPO, TIM).

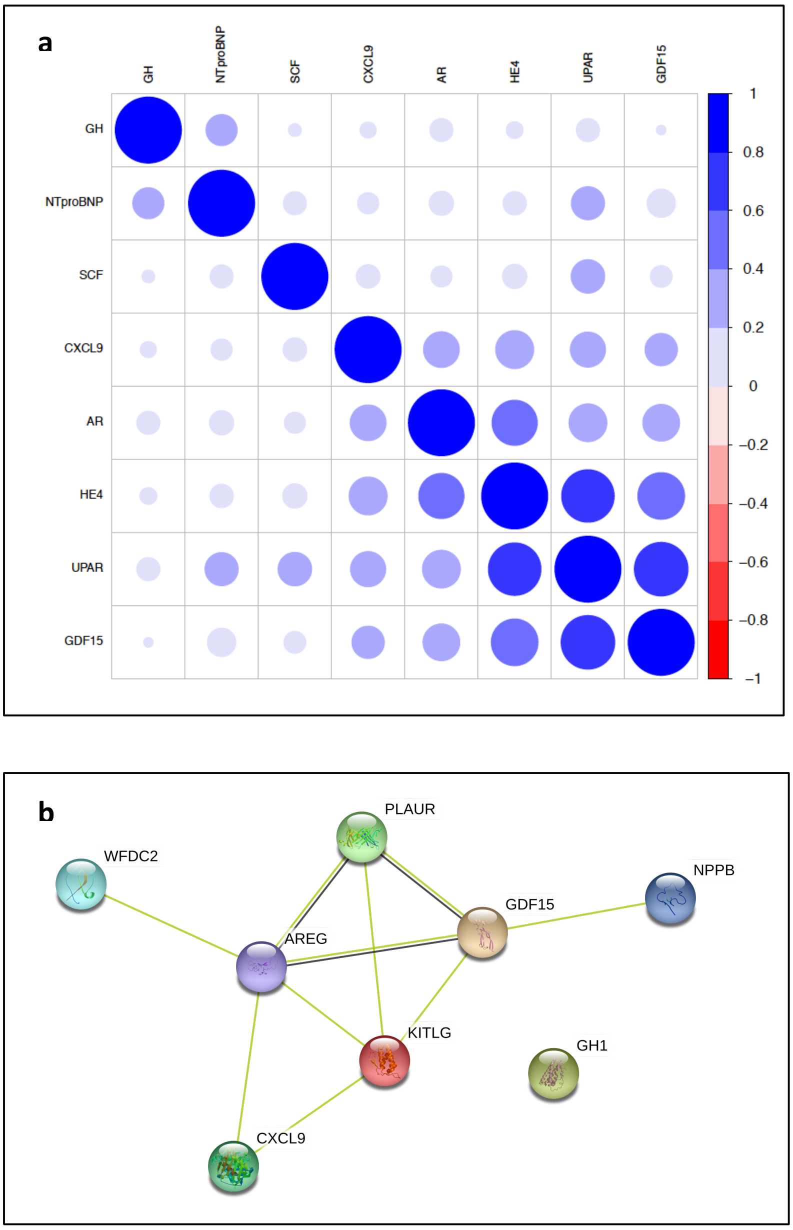

Supplement Figure 8. Heat map of Pearson correlation coefficients (**a**) and pathway analysis (**b**) for the top eight proteins associated with all-cause mortality in the MDCS-CVA. The pathway analysis was performed using the STRING database Version 11.0. Nodes represent proteins and edges represent protein-protein associations. All sources of interactions were probed and minimum required interaction score was set at low confidence (0.150). No known interactions (curated or experimental) were identified. Green edges represent connections by textmining and black edges represent putative co-expression. Abbreviations from the STRING database were used in the pathway analysis. Abbreviations: AREG – amphiregulin (AREG), WFDC2 – epididymal secretory protein E4 (HE4), PLAUR – urokinase plasminogen activator surface receptor (UPAR), GH1 – growth hormone (GH), NPPB – N-terminal pro-B-type natriuretic peptide (NTproBNP), CXCL9 – C-X-C motif chemokine 9 (CXCL9), KITLG – stem cell factor (SCF), GDF15 – growth/differentiation factor 15 (GDF15).

Supplement Table 5. Summary of proteins associated with all-cause mortality in two random samples of the MDCS-CVA using different methods. Proteins associated with all-cause mortality in only one random sample are denoted (+) and proteins associated with all-cause mortality in both random samples are denoted (++). All analyses adjusted for age, sex, smoking status, BMI, educational level, history of hypertension, prevalent diabetes mellitus, C-reactive protein (ln-transformed), HbA1c (ln-transformed), and LDL-cholesterol.

**Protein name (abbreviation)**	**Single protein**	**Stepwise Cox**	**Lasso Cox**	**RSF**
Adrenomedullin (AM)		+	+	
Agouti-related protein (AGRP)				
Amphiregulin (AR)	++		+	+
Angiopoietin-1 receptor (TIE2)				
B-cell activating factor (BAFF)				
C-C motif chemokine 19 (CCL19)	+	+	+	
C-C motif chemokine 20 (CCL20)				
C-C motif chemokine 3 (CCL3)				
C-C motif chemokine 4 (CCL4)			+	
C-X-C motif chemokine 1 (CXCL1)				
C-X-C motif chemokine 10 (CXCL10)				+
C-X-C motif chemokine 11 (CXCL11)	+	+		+
C-X-C motif chemokine 13 (CXCL13)			+	+
C-X-C motif chemokine 5 (CXCL5)				
C-X-C motif chemokine 6 (CXCL6)				
C-X-C motif chemokine 9 (CXCL9)	++	+	++	+
C-X-C motif chemokine-16 8CXCL16)				
Cadherin-3 (CDH3)				
Carbonic anhydrase IX (CAIX)				
Caspase-3 (CASP3)		++		
Caspase-8 (CASP8)				
Cathepsin D (CTSD)				+
Cathepsin L1 (CTSL1)				
CD40 ligand (CD40L)		+		+
Chitinase-3-like protein (CHI3L1)	+			+
Cyclin-dependent kinase inhibitor 1A (CDKN1A)				
Cystatin-B (CSTB)				
Dickkopf-related protein-1 (Dkk1)				
E-selectin (SELE)			+	
Early activation antigen CD69 (CD60)				
Endothelial cell-specific molecule 1 (ESM1)				
Eosinophil cationic protein (ECP)				
Epidermal growth factor (EFG)				
Epidermal growth factor receptor (EGFR)	+	++	++	+

Epididymal secretory protein E4 (HE4)	++	++	++	++
Epithelial cell adhesion molecule (EpCAM)				
Eukaryotic translation initiation factor 4B (eIF4B)				
Extracellular matrix metalloproteinase inducer (EMMPRIN)		+	+	+
Ezrin (EZR)		++	++	
Fas antigen ligand (FasL)	+		+	++
FAS-associated death domain protein (FADD)			+	
Fatty acid-binding protein, adipocyte (FABP4)				++
Fibroblast growth factor 23 (FGF23)	+			
Fms-related tyrosine kinase 3 ligand (Flt3L)				+
Folate receptor alpha (FRalpha)				
Follistatin (FS)			+	
Fractalkine (CX3CL1)				
Furin (FUR)				
Galanin peptides (GAL)			+	
Galectin-3 (Gal3)			+	+
Growth hormone (GH)	++		++	+
Growth/differentiation factor 15 (GDF15)	++	++	++	++
Heat shock 27 kDa protein (HSP27)			+	
Heparin-binding EGF-like growth factor (HBEGF)				
Hepatocyte growth factor (HGF)				++
ICOS ligand (ICOSLG)		+	+	
Immunoglobulin-like transcript 3 (ILT3)				
Integrin alpha-1 (ITGA1)		+		+
Interleukin-1 receptor antagonist protein (IL1ra)				+
Interleukin-12 (IL12)		+		++
Interleukin-16 (IL16)			+	
Interleukin-17 receptor B (IL17RB)	+	+	+	
Interleukin-18 (IL18)			+	
Interleukin-27 subunit alpha (IL27A)				+
Interleukin-6 (IL6)	++		+	++
Interleukin-6 receptor subunit alpha (IL6RA)				
Interleukin-7 (IL7)				
Interleukin-8 (IL8)				+
Kallikrein-11 (hK11)				+
Kallikrein-6 (KLK6)	+	+	++	+
Latency-associated peptide transforming growth factor beta-1 (LAPTGFbeta1)				
Lectin-like oxidized LDL receptor (LOX1)	+			
Leptin (LEP)				+
Macrophage colony-stimulating factor-1 (CSF1)				
Matrix metalloproteinase-1 (MMP1)				++

Matrix metalloproteinase-10 (MMP10)		+	+	
Matrix metalloproteinase-12 (MMP12)				++
Matrix metalloproteinase-3 (MMP3)				+
Matrix metalloproteinase-7 (MMP7)				
Melanoma-derived growth regulatory protein (MIA)				
Melusin (ITGB1BP2)				+
Membrane-bound aminopeptidase P (mAmP)				++
MHC class I polypeptide-related sequence A (MICA)	+	+	+	+
Midkine (MK)				+
Monocyte chemotactic protein 1 (MCP1)			+	
Myeloid differentiation primary response protein MyD88 (MYD88)				++
Myeloperoxidase (MPO)	+	+	+	+
Myoglobin (MB)	+		++	+
N-terminal pro-B-type natriuretic peptide (NTproBNP)	++	++	++	++
NF-kappa-B essential modulator (NEMO)			+	+
NT-3 growth factor receptor (NTRK3)				
Osteoprotegerin (OPG)				
Ovarian cancer-related tumor marker CA 125 (CA125)	+	+		+
P-selectin glycoprotein ligand 1 (PSGL1)				++
Pappalypsin-1 (PAPPA)				+
Parkinson protein 7 (PARK7)				
Pentraxin-related protein PTX3 (PTX3)				++
Placenta growth factor (PlGF)		+		
Platelet endothelial cell adhesion molecule (PECAM1)				
Platelet-derived growth factor subunit B (PDGFsubunitB)				
Prolactin (PRL)			+	
Prostasin (PRSS8)	+			++
Proteinase-activated receptor 1 (PAR1)				
Proto-oncogene tyrosine-protein kinase Src (SRC)				+
Receptor for advanced glycosylation end products (RAGE)			+	++
Receptor tyrosine-protein kinase erbB-2 (ErbB2HER2)				
Receptor tyrosine-protein kinase erbB-3 (ErbB3HER3)	+	+		
Receptor tyrosine-protein kinase erbB-4 (ErbB4HER4)				+
Regenerating islet-derived protein 4 (REG4)				
Renin (REN)				++
Resistin (RETN)				
SIR2-like protein-2 (SIRT2)		+		
Spondin-1 (SPON1)				
ST2 protein (ST2)		+		
Stem cell factor (SCF)	++		++	++
Tartrate-resistant acid phosphatase type 5 (TRAP)				

Thrombomodulin (TM)				
Thrombopoietin (THPO)				++
Tissue injury molecule-1/KIM-1 (TIM)	+	+	++	++
Tissue factor (TF)				
Tissue-type plasminogen activator (tPA)		+		
TNF-related activation-induced cytokine (TRANCE)				
TNF-related apoptosis-inducing ligand (TRAIL)	+	+	++	
TNF-related apoptosis-inducing ligand receptor 2 (TRAILR2)	+			+
Transforming growth factor alpha (TGFalpha)	+		+	
Tumor necrosis factor ligand superfamily member 14 (TNFSF14)				
Tumor necrosis factor receptor 1 (TNFR1)				
Tumor necrosis factor receptor 2 (TNFR2)		+		+
Tumor necrosis factor receptor superfamily member 4 (TNFRSF4)				+
Tumor necrosis factor receptor superfamily member 5 (CD40)				
Tumor nectorsis factor receptor superfamily member 6 (FAS)				
Tyrosine-protein kinase Lyn (LYN)				
Tyrosine-protein phosphatase, non-receptor type 22 (PTPN22)				++
Urokinase plasminogen activator surface receptor (UPAR)	++	+	++	+
Vascular endothelial growth factor A (VEGFA)				
Vascular endothelial growth factor D (VEGFD)				+
Vascular endothelial growth factor receptor 2 (VEGFR2)	+		+	
Vimentin (VIM)				+

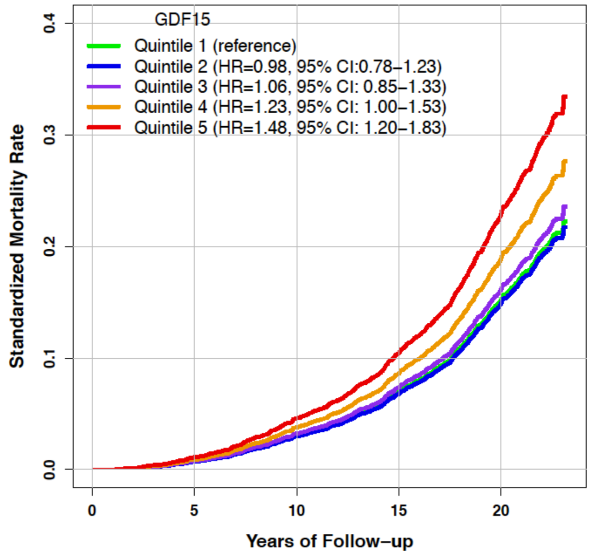

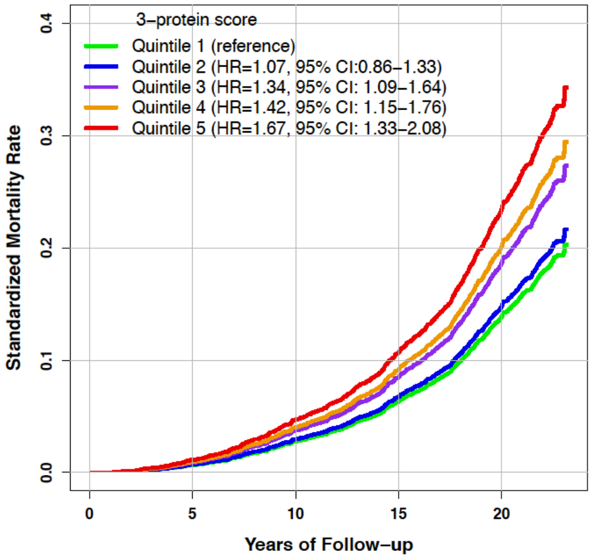

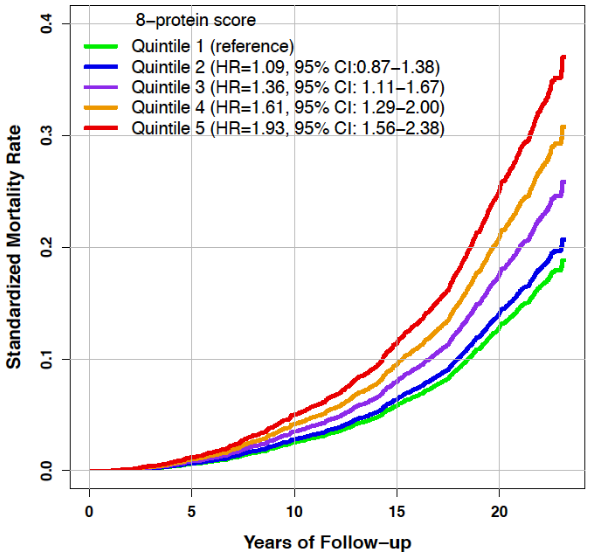

Supplement Figure 9. Standardized cumulative hazard plots of all-cause mortality by quintiles of protein scores in the MDCS-CVA (N=3,918). Cox regression models were adjusted for age, sex, smoking status, BMI, educational level, history of hypertension, prevalent diabetes mellitus, C-reactive protein, HbA1c, and LDL-cholesterol.

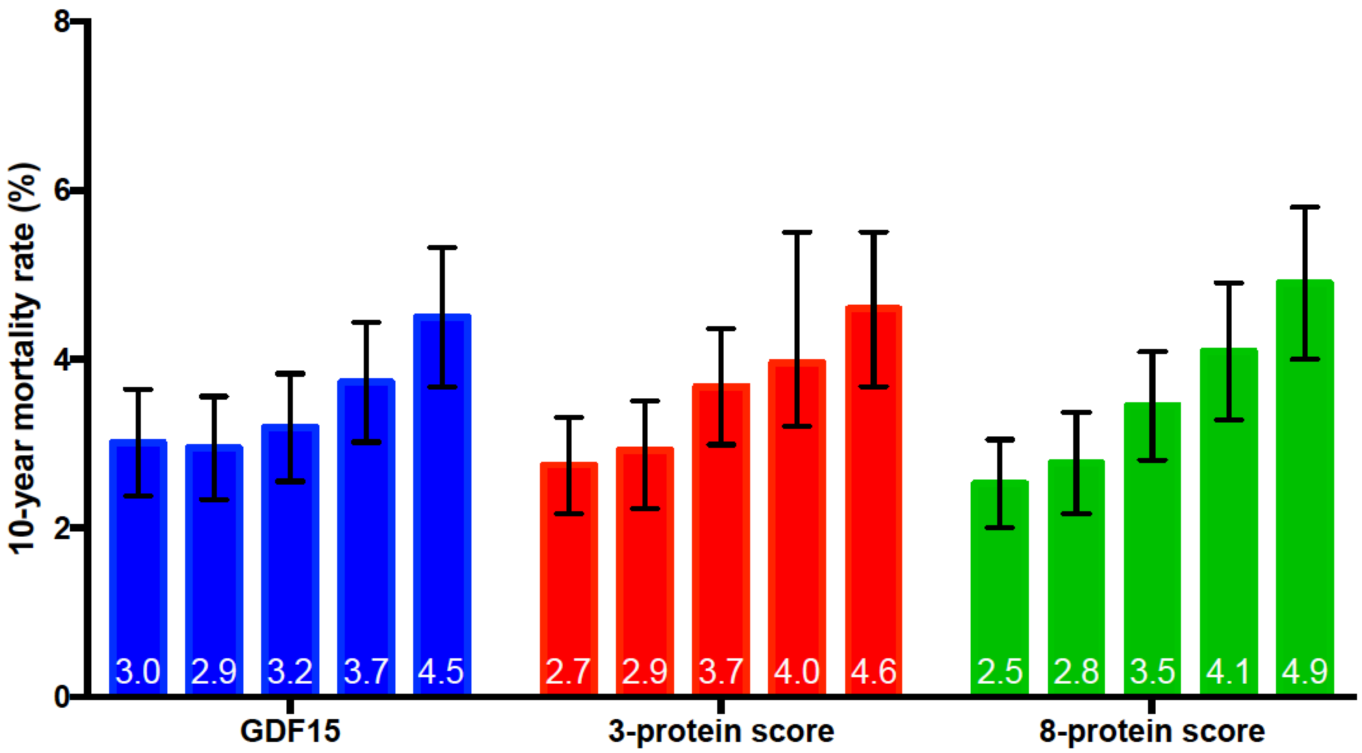

Supplement Figure 10. Standardized 10-year risk of all-cause mortality (%) across quintiles of protein scores in the MDCS-CVA (N=3,918). Absolute risk estimates adjusted for age, sex, smoking status, BMI, educational level, history of hypertension, prevalent diabetes mellitus, C-reactive protein, HbA1c, and LDL-cholesterol.
